# Sperm surface protein disulfide isomerase ERp57 is crucial for mammalian fertilization but functions independently of IZUMO1

**DOI:** 10.1186/s11658-026-00984-y

**Published:** 2026-06-27

**Authors:** Emily Forster, Sophie Dupuis, Côme Ialy-Radio, Vitor Hugo B. Serrão, Patrick Yip, Marine Foritano, Sandrine Barbaux, Jeffrey E. Lee, Ahmed Ziyyat

**Affiliations:** 1 Department of Laboratory Medicine and Pathobiology, Temerty Faculty of Medicine, University of Toronto, Toronto, ON M5S 1A8 Canada; 2 Université de Paris, Institut Cochin, INSERM, CNRS, 75014 Paris, France; 3 Service d’Histologie, d’Embryologie, Biologie de La Reproduction, AP-HP, Hôpital Cochin, 75014 Paris, France; 4 Current Address: Biomolecular Cryo-Electron Microscopy Facility, University of California-Santa Cruz, Santa Cruz, CA 95064 USA

**Keywords:** Fertilization, Sperm-egg fusion, ERp57, PDIA3, Protein disulfide isomerase, IZUMO1, JUNO, Membrane fusion

## Abstract

**Background:**

Human fertilization requires fusion of spermatozoon and oocyte membranes to form a diploid zygote, beginning with adhesion mediated by spermatozoon IZUMO1 and oocyte JUNO. Current models propose that IZUMO1 dimerizes after interacting with JUNO, possibly triggered by a protein disulfide isomerase. It has been proposed that protein disulfide isomerase ERp57 is the trigger for IZUMO1 dimerization, a mechanism supported by parallels in viral entry, but direct evidence is lacking.

**Methods:**

In vitro fertilization studies were performed for both mice and humans using ERp57 inhibitors to confirm the importance of ERp57 in mammalian fertilization. Additionally, for this study, we generated a sperm-specific *ERp57* conditional knockout mouse model and performed in vivo and in vitro fertilization experiments. Biophysical assays, including dynamic light scattering and a fluorescence-based dissociation assay, were developed and utilized to investigate interactions between ERp57 and IZUMO1. Structural modeling was used to supplement the ERp57 and IZUMO1 interaction findings.

**Results:**

Here, we reveal that ERp57 is crucial for mammalian fertilization but does not show evidence of any direct interaction with IZUMO1. ERp57 inhibition significantly reduces fertilization in human and mouse in vitro assays, and *ERp57* spermatozoa conditional knockout (scKO) males exhibit severe hypofertility in vivo and in vitro. ERp57 localizes to the equatorial segment of human spermatozoa following the acrosome reaction, consistent with a role in gamete interaction. However, ERp57-deficient spermatozoa fail to accumulate in the perivitelline space, pointing to a role upstream of membrane fusion. Additionally, ERp57 neither promotes IZUMO1 dimerization nor facilitates dissociation of the IZUMO1-JUNO complex. Structural modeling predicted no significant interaction between ERp57 and IZUMO1, supporting experimental findings.

**Conclusions:**

These findings establish ERp57 as critical for mammalian fertilization but challenge existing assumptions about its mechanistic involvement in gamete membrane fusion. Our research contributions provide key new mechanistic insights that reexamine and reshape the current paradigms surrounding the fundamental process of sperm-egg fusion. By addressing a long-standing bottleneck in the field, our work opens new avenues of investigation that could finally lead to the identification of the elusive human sperm-egg fusogen.

**Supplementary Information:**

The online version contains supplementary material available at https://doi.org/10.1186/s11658-026-00984-y.

## Background

Reproduction in mammals requires the fusion of two haploid gametes, a spermatozoon and an oocyte, to form a diploid zygote, a process known as sperm-egg fusion. Fertilization begins with capacitation as spermatozoa travel through the female reproductive tract, undergoing membrane reorganization that exposes surface molecules important for guiding spermatozoa towards the oocyte. The spermatozoa then traverse the cumulus cells surrounding the oocyte and establish initial binding with zona pellucida (ZP) glycoproteins. This ZP interaction triggers the acrosome reaction, if it has not already occurred, during which the acrosomal membrane fuses with the spermatozoon plasma membrane, releasing acrosomal enzymes that digest components of the extracellular matrix around the oocyte. Acrosome-reacted spermatozoa can then penetrate the ZP and enter the perivitelline space (PVS). Finally, adhesion and subsequent fusion between the spermatozoon and oocyte membranes occur, allowing the combination of their genetic material [1–4]. Several protein factors have been identified to be critical for mammalian sperm-egg adhesion/fusion [4]. On the spermatozoa side, these are IZUMO1 [5–8], SPACA6 [9, 10], FIMP [11], SOF1 [10], TMEM95 [10, 12], TMEM81 [13], DCST1 and DCST2 [14]. IZUMO1, SPACA6 and TMEM81 form a complex on the surface of spermatozoa [13]. On the oocyte side, there are known tetraspanins CD9 [15–17] and CD81 [18] as well as the glycosylphosphatidylinositol-anchored protein JUNO [19], which is the binding partner of IZUMO1. IZUMO1 and JUNO represent the only known essential binding pair for human gamete adhesion [2, 5–7, 19].

Inoue et al*.* first proposed a schematic model where monomeric IZUMO1 and JUNO bind to each other during gamete recognition followed by a transition where IZUMO1 dimerizes [20]. It was suggested that the action of a protein disulfide isomerase (PDI) modifies the disulfide bridges of IZUMO1 to structurally convert to a closed dimer form. The IZUMO1 dimerization is then thought to lead to JUNO release from the oocyte surface to open sites for potential recruitment of new protein factors for fusion of the spermatozoon and oocyte membranes [20, 21]. Recombinant IZUMO1 dimers have even been observed in solution [22, 23], with indication that its helical dimerization is required for successful fusion [23]. However, this fusion model has not been proven experimentally, as the triggering PDI and downstream functions of IZUMO1 have not been identified.

While PDIs typically reside in the endoplasmic reticulum (ER) to assist with post-translational protein folding, there are three PDI family members, PDIA1 (PDI), PDIA3 (ERp57), and PDIA6 (P5), on the surface of spermatozoa [24]. Inhibition of sperm-egg fusion in vitro has been observed using different inhibitors of PDI activity [25]. Antibodies against ERp57, but not PDI and P5, were able to decrease in vitro fertilization (IVF) in mice [25]. Though a more recent study shows that P5 conditional knockout mice are infertile, this is most likely explained by their abnormal spermatozoa morphology, impaired acrosome reaction, and increased apoptosis [26]. Relocation of ERp57 on the human spermatozoon surface after the acrosome reaction suggested a role in gamete adhesion and/or membrane fusion [27]. Moreover, there is strong inhibition of fusion between human spermatozoa and hamster oocytes in the presence of an antibody directed against human ERp57 (hERp57) [27]. Since ERp57 is known to be present on the spermatozoon membrane and appears involved in fertilization, this has been connected back to the idea that IZUMO1 structural change and JUNO shedding could be triggered by this PDI. The proposed mechanism of ERp57 as the PDI trigger for IZUMO1 dimerization is one of the main hypotheses in the field [2, 20], though there is no direct experimental evidence to support this.

In this study, our goal was to address two key questions: Is ERp57 involved in mammalian fertilization, and does it play a role in triggering IZUMO1 to adopt its closed dimeric conformation, as proposed by Inoue et al. [20]? Our findings demonstrate the impact of PDI inhibitors and anti-hERp57 antibodies on IVF. Specifically, we observed a significant reduction in the fertilization index in human IVF assays in the presence of anti-hERp57 antibodies. Furthermore, a conditional knock-out (KO) mouse line lacking the *ERp57* gene specifically in spermatozoa displayed severe hypofertility in males in vivo. Its spermatozoa did not accumulate in the PVS and showed a near-complete inability to fertilize oocytes in vitro. These results firmly establish the critical role of ERp57 in mammalian fertilization. However, through a variety of biochemical and computational methods, we found that hERp57 does not directly interact with IZUMO1 or the IZUMO1-JUNO complex to induce oligomerization. This changes the current narrative in the field regarding the role of human ERp57 in sperm-egg fusion.

## Materials and methods

### Generation and breeding of transgenic mice

Mice with a conditional deletion of *ERp57* in the spermatozoa (scKO) were generated by crossing *ERp57* floxed mice (provided by Prof. Vikas Anathy, University of Vermont and created by Günter J Hämmerling from German Cancer Research Center, Heidelberg, Germany, as described by Garbi et al*.* [28]) with mice expressing Cre recombinase under the control of a 1.4 kb promoter region of the germ cell-specific *Stimulated by retinoic acid gene 8* (*Stra8*) (JAX stock #017490) [29]. Homozygous floxed *ERp57* mice and homozygous *Stra8-cre* mice were first crossed to produce double heterozygous offspring. From this stage, two alternative scenarios are possible with several possible male and female genotypes at each cross.

In the first scenario, the obtained females (*ERp57 WT/fl Stra8-cre (* +/-*)*) were crossed with homozygous males (*ERp57 fl/fl Stra8-cre (-/-)*) resulting in several genotypes, two of which are of interest: scKO males (*ERp57 fl/fl Stra8-cre (* +/-*)*) and their littermate male controls (*ERp57 fl/fl Stra8-cre (-/-)*). Cre expression in the male germline of these double-floxed males would allow the production of a majority of excised spermatozoa.

In the second scenario, scKO males (*ERp57 fl/del Stra8-cre (* +/-*)*) already carrying an excised copy of *ERp57* in their somatic DNA were generated. This configuration should be more efficient to excise the second remaining floxed allele. These males could be obtained by the crossing between double heterozygous males expressing Cre in their germline (*ERp57 WT/fl Stra8-cre (* +/-*)*) and homozygous floxed females *(ERp57 fl/fl Stra8-cre (-/-)*). Control littermate males (*ERp57 fl/del Stra8-cre (-/-)*) maintained one floxed allele and one deleted allele since they did not express Cre. These latter scKO males that theoretically reach the highest level of excision, along with their controls, were used in this study for in vivo and in vitro fertility assays.

Genotyping of scKO males was performed by PCR amplification on DNA extracted from tail biopsies and spermatozoa (NucleoSpin Tissue, Macherey–Nagel, Düren, Germany) using the GoTaq Flexi DNA polymerase (Promega, Madison, WI, USA) under standard PCR conditions. Specific primers were used as follows: Primer Up1 (in intron 1–2 of *ERp57*; 5′-GGAATGCCCTGTAATGTCACTATG-3′) and primer R1 (in exon 2 of *ERp57*; 5′-GGCAAGCCTCTTGCAATGTCCACA-3′) detect the floxed allele, while primer F1 (in intron 1–2 of *ERp57*; 5′-CGCCAGCCTCTCCATTTAGAGAGA-3′) and primer R2 (in exon 4 of *ERp57*, 5′-TGAAGCTGGTCCTGCTTGTTTC-3′) detect the deleted allele (Eurogentec, Liege, Belgium), and primer Up6 (in intron 2–3; 5’-ATGTGAGTCCTTAGGCTTAGCA-3’) and Dn6 (in intron 2–3; 5’-TTGCCCCTTGGTTCTTATTCAC-3’) detect the remaining portion of the sequence between the two LoxP sites. Primers F3 (5′-AGATGCCAGGACATCAGGAACCTG-3′) and R3 (5′-ATCAGCCACACCAGACACAGAGATC-3′) were used to detect the presence of the *Stra8*-*cre*. A PCR product of about 700 bp was observed for the WT *ERp57* allele and about 1000 bp for the floxed *ERp57* allele with primers Up1-R1. A positive amplification between primers F1 and R2 (~ 850 bp) indicates a deletion between the two LoxP site in *ERp57*. The expression of the *Stra8*-*cre* was detected when a PCR product of 236 bp was obtained with primers F3 and R3. PCR products were sequenced (Eurofins Genomics, Les Ulis, France) to confirm deletion and Cre expression. On spermatozoa DNA from scKO and controls, Up1-R1 primers were used in semi-quantitative PCR, of 15, 20, and 25 cycles, demonstrating that the remaining floxed (non-excised) allele appeared early in the control compared to the scKO males, and confirming that there are still some floxed alleles left in the spermatozoa of scKO males.

### Mouse in vivo fertility

Sexually mature scKO male and control male littermates were mated with 7-week-old C57BL/6J females (Janvier Labs, Le Genest-Saint-Isle, France). The numbers of pups and litters were recorded after 3 weeks of gestation, with mating confirmed by the presence of a vaginal plug. For the fertilization assay, 5- to 8-week-old WT C57BL/6J female mice were injected with 5 IU pregnant mare serum gonadotropin (PMSG), followed 48 h later with 5 IU human chorionic gonadotropin (hCG) (Intervet, Beaucouzé, France) to induce superovulation. Females were then mated overnight with pubertal control or scKO males to evaluate their capacity to fertilize in vivo. The next day, females with a vaginal plug were sacrificed by cervical dislocation, and MII-oocytes were retrieved. For simplicity, MII-oocytes are hereafter referred to as oocytes. Cumulus cells were removed, and oocytes were directly mounted in Vectashield/DAPI (Vector laboratories, Burlingame, CA, USA) for observation on a Nikon Eclipse E600 UV microscope. Oocytes containing at least one fluorescent decondensed spermatozoon head within their cytoplasm were considered fertilized, and the fertilization rate (FR) was calculated based on this criterion.

### Mouse in vitro fertilization assay

#### Oocytes preparation

WT C57BL/6J female mice (for in vivo and in vitro experiments with scKO males) and 5- to 8-week-old WT B6CBAF1 female mice (for IVF experiments with inhibitors and antibodies) (Janvier Labs, Le Genest-Saint-Isle, France) were superovulated with 5 IU PMSG, followed 48 h later by addition of 5 IU hCG (Intervet, Beaucouzé, France). Approximately 13 h after hCG injection, animals were sacrificed by cervical dislocation. Cumulus-oocyte complexes (COC) were collected by gently tearing the ampulla wall of the oviduct and then placed in FertiCult medium (FertiPro N.V, Belgium) supplemented with 3% (w/v) BSA (Sigma–Aldrich). The COC were maintained at 37 °C under 5% CO_2_ under FertiCult mineral oil (FertiPro N.V, Belgium). For experiments involving zona-free oocytes, cumulus cells were removed by brief exposure to hyaluronidase IV-S (1 mg/ml, Sigma–Aldrich), and the ZP was dissolved using acidic Tyrode’s solution (pH 2.5, Sigma–Aldrich), under visual monitoring. Zona-free oocytes were rapidly washed in FertiCult medium, 3% (w/v) BSA, and incubated at 37 °C in 5% CO_2_ atmosphere for 2–3 h to recover their fertilization capacity.

#### Spermatozoa preparation

Mouse spermatozoa were collected from the cauda epididymis of 8-week-old C57BL/6 J control or scKO male mice (for in vivo and in vitro experiments) and B6CBAF1 WT males (for IVF experiments with inhibitors and antibodies). Spermatozoa were capacitated for 90 min at 37 °C in a 500 μl drop of FertiCult medium with 3% (w/v) BSA under FertiCult mineral oil, in a 5% CO_2_ atmosphere. Spermatozoa samples were then pre-incubated with or without the inhibitors: bacitracin (1.5 mM or 2 mM), punicalagin (60 μM or 80 μM), 100 μg/mL mouse anti-hERp57 antibody (Santa Cruz Biotechnology, clone 4E69) or 100 μg/mL IgG1 isotype control antibody (BioLegend, clone MG1-45) for an additional 30 min at 37 °C in FertiCult medium with 3% (w/v) BSA under 5% CO_2_.

#### In vitro fertilization

Cumulus-intact or zona-free oocytes were inseminated with capacitated spermatozoa at a final concentration of 1 × 10^6^ cells/mL or 1 × 10^5^ cells/mL, respectively. Spermatozoa could be provided by scKO or their control males or by WT males. In this latter case, spermatozoa were pre-incubated with or without inhibitors or antibodies. Insemination occurred for 3 h in a 50 μL drop of FertiCult medium with 3% (w/v) BSA maintained at 37 °C, 5% CO_2_, under FertiCult mineral oil. After incubation, oocytes were washed and directly mounted in Vectashield/DAPI and visualized using a Nikon Eclipse E600 UV microscope. Oocytes containing at least one fluorescent decondensed spermatozoon head in their cytoplasm were considered fertilized, and the FR was calculated. For the zona-free IVF assay, the fertilization index (FI) was determined by counting the number of decondensed spermatozoon heads per oocyte.

### Human in vitro fertilization assay

In vitro maturated, unfertilized human MII-oocytes were used for IVF assays. These experiments were performed with depellucidated (zona-free) oocytes, which were obtained by chemically dissolving the ZP using acidic Tyrode’s solution (pH 2.5,) under visual monitoring. Zona-free oocytes were washed several times in FertiCult medium with 3% (w/v) BSA and incubated at 37 °C under 5% CO_2_ in FertiCult mineral oil for 2 h to recover their fertilization capacity. Spermatozoa samples were capacitated for 3 h at 37 °C in FertiCult medium supplemented with 3% (w/v) BSA under 5% CO_2_ and FertiCult mineral oil. The spermatozoa were then pre-incubated with either anti-hERp57 (Santa Cruz Biotechnology, clone 4E69) or IgG1 isotype control antibodies (20 μg/mL), or without antibodies for 30 min at 37 °C and 5% CO_2_. Zona-free oocytes were inseminated with capacitated spermatozoa at a final concentration of 1 × 10^5^ cells/mL for 18 h at 37 °C in 5% CO_2_ in a 20 μL drop of FertiCult medium with 3% (w/v) BSA containing either mouse anti-hERp57 antibody (20 μg/mL), mouse IgG1 isotype control (20 μg/mL) or no antibody. After incubation, oocytes were washed and mounted using Vectashield Mounting Medium with DAPI for visualization using a Nikon Eclipse E600 UV microscope. Zona-free oocytes with a fluorescent, decondensed spermatozoon head within their cytoplasm were considered fertilized. The FI was calculated by counting the number of decondensed spermatozoon heads per oocyte.

### Spermatozoa ERp57 analysis

#### Mouse spermatozoa immunostaining

Mouse spermatozoa samples were capacitated at 37 °C under 5% CO_2_ for 3 h in FertiCult medium supplemented with 3% (w/v) BSA, under mineral oil. Following capacitation, spermatozoa were washed in 1X PBS containing 1% (w/v) BSA, centrifuged at 600 × *g* for 5 min, and immediately fixed in 4% (w/v) paraformaldehyde (Electron Microscopy Sciences, PA, USA) for 10 min at room temperature (RT). The spermatozoa were then washed again in 1X PBS-1% (w/v) BSA and permeabilized using a 1X PBS-0.2% (v/v) Tween-20 solution. After three washes, samples were blocked for 30 min in 1X PBS-3% (w/v) BSA at RT. For immunostaining, spermatozoa were incubated 2 h at 37 °C with anti-hERp57 antibody (Santa Cruz Biotechnology, clone 4E69) at 5 μg/mL or, with mouse IgG1 isotype control antibody (BioLegend Inc, clone MG1-45) at 5 µg/mL in 1X PBS-1% (w/v) BSA. After three additional washes in 1X PBS-1% (w/v) BSA, the spermatozoa were incubated with an anti-mouse Alexa Fluor 488 secondary antibody at 5 μg/ml for 1 h at RT. In order to assess the acrosomal status, spermatozoa were stained with 10 µg/mL rhodamine-conjugated Pisum Sativum Agglutinin (Vector Laboratories, Burlingame, CA, USA) for 15 min. Following three washes in 1X PBS-1% (w/v) BSA, a drop of spermatozoa suspension was smeared on a slide, air-dried, and mounted using Vectashield Mounting Medium with DAPI. The samples were visualized using a Leica spinning disk confocal microscope.

#### Human spermatozoa immunostaining

Human spermatozoa samples were initially washed in FertiCult medium with 3% (w/v) BSA, followed by overnight incubation with 13 µg/mL anti-IZUMO1 nanobody (ZNb1, custom generated by Lee Lab) and 7 µg/mL anti-hERp57 antibody (Santa Cruz Biotechnology, clone 4E69) in FertiCult medium with 3% (w/v) BSA. For the negative control, spermatozoa samples were incubated with 7 µg/mL of mouse IgG1 κ isotype control (BioLegend Inc, clone MG1-45). The following day, samples were washed three times in FertiCult medium with 3% (w/v) BSA and incubated for 1 h at 37 °C under 5% CO_2_ with secondary antibodies: anti-mouse Alexa Fluor 488 (ThermoFisher Scientific, A-21200) at 10 μg/mL, and goat anti-alpaca IgG Alexa Fluor 594 AffiniPure, VHH domain (Jackson ImmunoResearch, UK, 128–585-232) at 1.7 µg/mL. After repeated washing with FertiCult medium, 3% (w/v) BSA, spermatozoa samples were fixed in 4% (w/v) paraformaldehyde in 1X PBS for 5 min, followed by three washes with 1X PBS-1% (w/v) BSA. A drop of spermatozoa suspension was smeared on a slide, air-dried and mounted with the Vectashield Mounting Medium with DAPI. Detection was performed using a Leica spinning disk confocal microscope.

#### ERp57 antibody Western blot expression analysis

Spermatozoa (human, WT or scKO males) pellets were extracted using a 2% (w/v) SDS, 1X PBS solution supplemented with protease inhibitors. Recombinant mouse ERp57 protein was obtained from Abcam (ab222976), and recombinant human protein was expressed in-house, as described below. Proteins were run, in non-denaturing conditions for human and mouse samples, on a precast Mini-Protean TGX gel (Bio-Rad, Marnes la Coquette, France), transferred to a nitrocellulose membrane and probed with the previously mentioned mouse anti-hERp57 antibody.

### Recombinant protein preparation

#### Human ERp57

DNA corresponding to the human *ERp57* gene (residues 25–505; Uniprot P30101) was codon optimized for *E. coli* expression, gene synthesized and subcloned into pET46 Ek/LIC according to manufacturer instructions. The recombinant hERp57 contains a 6X His tag at the N-terminus followed by an enterokinase and thrombin cleavage sites (MAHHHHHHVDDDDKLVPRGS-hERp57 sequence). For the hERp57 double mutant, four-point mutations (C57A, C60A, C406A and C409A) were introduced by QuikChange site-directed mutagenesis to mutate the catalytic site reactive cysteines. The hERp57-pET46 vector was transformed into *E. coli* BL21 (DE3) competent cells (Millipore Sigma/Novagen 69,450) by heat shock and a single colony was inoculated into a 20 mL LB overnight starter culture supplemented with final concentration 100 µg/mL ampicillin and grown at 37 °C in an orbital shaker at 180 rpm. 1L LB culture with 100 µg/mL ampicillin final concentration was inoculated with 10 mL LB starter culture and grown to an optical density (OD600) of 0.80 at 37 °C and 180 rpm. Cells were then induced for expression with a final concentration of 1 mM isopropyl β-D-1-thiogalactopyranoside (IPTG) and grown at 37 °C for 4 h. Cells were harvested by centrifugation at 2,700 × *g* for 45 min and either lysed immediately or frozen at -20 °C. For lysis, cells were resuspended in 30 mL Lysis Buffer (50 mM Tris–HCl pH 8.0, 300 mM NaCl, 20 mM imidazole) and lysed using a hydraulic cell disruption system (Constant Systems CF1 TS 0.75) at 30 kpsi. The resulting lysate was sonicated 3 × 30 s on ice (power setting = 4) to reduce sample viscosity by shearing nucleic acid. Cell lysate was then clarified by centrifugation at 4 °C for 45 min at 17,600 × *g.* The supernatant was applied to a 4 mL Ni–NTA column (Qiagen) equilibrated with Lysis Buffer. The column was washed thrice with 5 CV Lysis Buffer, and WT and double mutant hERp57 were each eluted in 10 CV 50 mM Tris–HCl pH 8.0, 300 mM NaCl, 50 mM imidazole. Elution fractions were concentrated to 1 mL using an Amicon Ultra-15 Centrifugal Filter with 30 kDa MWCO (Millipore Sigma, UFC9030) and isocratically eluted from a Superdex 75 Increase 10/300 column equilibrated in 50 mM Tris–HCl pH 8.0, 150 mM NaCl, 1 mM dithiothreitol (DTT) and 5% (v/v) glycerol. Protein purity was determined by Coomassie-stained SDS-PAGE analysis and Western blot using mouse anti-hERp57 primary antibody (Santa Cruz Biotechnology, clone 4E69) and goat anti-mouse HRP-conjugated secondary antibody (Thermo Fisher, #62–6520). Peak fractions containing hERp57 were pooled, aliquoted, flash frozen in liquid nitrogen, and stored at -80 °C. Total protein was quantified by measuring A_280_. Protein purity was determined by electrospray ionization mass spectrometry (AIMS Mass Spectrometry Laboratory, University of Toronto, Department of Chemistry).

#### Human IZUMO1 and JUNO

Recombinant IZUMO1 and JUNO were expressed as described previously [5]. Briefly, DNA sequences encoding IZUMO_22-254_ and JUNO_20-228_ were codon optimized for *D. melanogaster* and cloned into the pMT expression vector modified with a puromycin selection marker. Both constructs included a thrombin cleavage site followed by a 10X His affinity tag at the C-terminus. The plasmids were transfected into Drosophila S2 cells (Invitrogen R69007) using Effectene transfection reagent (Qiagen). Stably transfected cells were selected by puromycin and slowly transitioned to Insect-XPRESS growth media (Lonza). Protein expression was induced with a final concentration 500 µM CuSO_4_. Cultured media was collected 4 days post-induction, concentrated and buffer exchanged using a Centramate tangential flow filtration system (Pall Corp). All IZUMO1 and JUNO proteins were purified by Ni–NTA metal affinity chromatography, followed by thrombin cleavage and size exclusion chromatography (SEC) using a Superdex 75 Increase 10/300 column equilibrated with HBS (10 mM HEPES-HCl pH 7.5, 150 mM NaCl) and 5% (v/v) glycerol. Peak fractions containing IZUMO1 or JUNO were pooled, aliquoted, flash frozen in liquid nitrogen, and stored at -80 °C. Total protein was quantified by measuring A_280_ and protein purity was determined by Coomassie-stained SDS-PAGE analysis.

#### IZUMO1-JUNO complex

Purified IZUMO1 and JUNO were mixed in an IZUMO1:JUNO molar ratio of 1.1 to 1 and incubated on ice for 1 h. The complex was then purified by SEC using a Superdex 75 Increase 10/300 column equilibrated with HBS and 5% (v/v) glycerol. The main peak was pooled, aliquoted, flash frozen in liquid nitrogen, and stored at -80 °C.

### Insulin turbidity assay

Enzymatic activity of hERp57 was assessed using an insulin turbidity assay [30, 31], which measures the increase in turbidity resulting from the reduction of the α- and β-chains of insulin. Reactions were performed in a 96-well clear flat bottom polystyrene microplate (Corning, 3370) with a total reaction volume of 200 µL. Bovine pancreas insulin (Sigma-Aldrich, I5500) was reconstituted in 50 mM Tris–HCl pH 7.5 at 10 mg/mL. For inhibition studies, bacitracin (Sigma-Aldrich, 11702) was dissolved in 1X PBS to a concentration of 40 mM and freshly prepared for each experiment, while punicalagin (Sigma-Aldrich, P0023) was dissolved in 100% (v/v) DMSO to 20 mM and used from a -80 °C stock protected from light. Each well contains 30 µL of enzyme/inhibitor mix and 150 µL of a freshly prepared insulin cocktail (1.33 mg/mL bovine pancreas insulin, 84 mM sodium phosphate buffer pH 7.0 and 2.67 mM sodium EDTA pH 7.0). The 30 µL enzyme/inhibitor mix contains 10 µg hERp57 WT or hERp57 double mutant with bacitracin or punicalagin. For punicalagin-treated samples and relevant controls, the final DMSO concentration was 2% (v/v). Prior to absorbance readings, 20 µL 10 mM DTT was added to each well. Absorbance at 650 nm was measured in 2 min intervals over 90 min using a Synergy Neo2 multimode microplate reader (BioTek/Agilent Technologies) at 25 °C with continuous orbital shaking. PDI storage buffer (without hERp57) served as a negative control, while bovine liver PDI (Sigma-Aldrich, P3818) was used as a positive control. Absorbance measurements were normalized by subtracting the value of the corresponding control without enzyme. All experiments were performed in technical triplicate and repeated three times.

### Sequence logo plot generation

88 mammalian IZUMO1 sequences listed in Supplementary Table S1 were retrieved from Uniprot [32] and aligned in Clustal Omega [33, 34]. The multiple sequence alignment (MSA) was used to generate the logo plots in WebLogo [35].

### Disulfide modifications of IZUMO1 and hERp57

IZUMO1, WT hERp57, or a combination of both were incubated in TBS (10 mM Tris–HCl pH 7.5, 150 mM NaCl) at 40 µM final concentration each in 80 µL for 2 h at RT. Maleimide (Sigma-Aldrich, 129585) was added to a final concentration of 400 µM and the samples were incubated for 25 min at RT, protected from light. Each sample was then buffer exchanged into low-salt TBS (50 mM NaCl) with at least 8 cycles of repeated concentration and dilution using Amicon Ultra-0.5 Centrifugal Filters with 10 kDa MWCO (Sigma-Aldrich, UFC5010). Electrospray ionization-mass spectrometry (ESI–MS) assays were performed at the AIMS Mass Spectrometry Laboratory (University of Toronto, Department of Chemistry).

### Dynamic light scattering-based oligomerization assay

Protein standards were prepared in 20 µL well volumes, each at a final concentration of 1.5 mg/mL: lysozyme (Sigma-Aldrich, 62971), carbonic anhydrase (Sigma-Aldrich, C7025-1VL), bovine serum albumin (Sigma-Aldrich, A3294), alcohol dehydrogenase (Sigma-Aldrich, A8656-1VL), thyroglobulin (Sigma-Aldrich, T9145-1VL). Samples were centrifuged at 20,000 × *g* for 15 min at 4 °C. Samples were then transferred to a 384-well black round bottom polystyrene microplate (Corning, 3540), with 20 µL total volume per well. The 384-well plate was centrifuged for 2 min at 400 × *g* before measurements were recorded on a Wyatt DynaPro Plate Reader II (Waters). Protein preparations of IZUMO1, IZUMO1-JUNO complex, or JUNO were prepared at 36 µM final concentration in TBS. HBS with 5% (v/v) glycerol, the buffer conditions in which the proteins were frozen, was used as a negative buffer control. A 1 mM DTT treatment of IZUMO1 served as a positive control. Protein and treatment samples were centrifuged for 15 min at 4 °C at 20,000 × *g*. 10 µL of protein sample was added to each well, followed by 10 µL of treatment sample. The plate was briefly spun, as described above, before readings were recorded every 30 min over 2.5 h. For the main experiment, hERp57 WT was prepared at 36 µM final concentration in TBS. PDI storage buffer (10 mM Tris–HCl pH 8.0, 150 mM NaCl, 1 mM DTT, 5% (v/v) glycerol) was used as a negative control. All samples were prepared in the same manner as the DTT control experiment. Each well contained a final concentration of 3% (v/v) glycerol and 300 µM DTT from protein and buffer components. The time from sample mixing to first reading was 10 min. Due to the speed of measurement of the instrument, a maximum of 24 samples were read per experiment.

### IZUMO1-JUNO dissociation assay

#### IZUMO1 biotin and JUNO FITC conjugation

EZ-Link Sulfo-NHS-LC-Biotin (ThermoFisher Scientific, A39257) was dissolved in water according to manufacturer instructions and added to IZUMO1 at a 1:1 molar ratio of biotin to protein. FITC isomer I (Sigma-Aldrich, F7250) was dissolved in DMSO to a final concentration of 50 mM and then added to JUNO at a 3:1 molar ratio of FITC to protein. The mixture was incubated on ice for 2 h in the dark. Excess biotin or FITC was removed by overnight dialysis at 4 °C against 4 L of HBS using SnakeSkin 10 kDa MWCO dialysis tubing (ThermoFisher Scientific, 88243). After dialysis, a final concentration of 50 mM Tris–HCl pH 7.5 was added to each labeled protein solution to halt further labeling. To form the complex, IZUMO1^biotin^ and JUNO^FITC^ were mixed at a molar ratio of 1 to 1.1 and incubated on ice for 1 h. The IZUMO1^biotin^-JUNO^FITC^ complex was subsequently purified by SEC at 4 °C on a Superdex 75 Increase 10/300 column protected from light and equilibrated with 1X HBS pH 7.5 and 5% (v/v) glycerol. Fractions containing the IZUMO1^biotin^-JUNO^FITC^ complex were pooled and concentrated to a final volume of ~ 60 µL using an Amicon Ultra-0.5 centrifugal filter (30 kDa MWCO; Millipore Sigma, UFC5030). The fluorescein-to-protein molar ratio and complex concentration were determined using A_495_ and A_280_, according to manufacturer protocol. The complex was aliquoted, flash frozen in liquid nitrogen, and stored at -80 °C.

#### JUNO dissociation assay

The dissociation assay was performed using a black 96-well Pierce Streptavidin Coated Plate (ThermoFisher Scientific, 15119) protected from light in all subsequent steps. Wells were washed three times with 200 µL of Wash Buffer (1X PBS pH 7.4, 0.1% (w/v) BSA, 0.05% (v/v) Tween-20). Next, 1 µg of IZUMO1^biotin^-JUNO^FITC^ (experimental) or IZUMO1^biotin^-JUNO (control) was added to each well in a total volume of 100 µL. The plate was incubated with complex for 2 h at RT, then washed twice with 200 µL of Wash Buffer. To block nonspecific binding, 200 µL of Wash Buffer was added to each well and incubated for 60 min at RT. After blocking, the Wash Buffer was replaced with the appropriate enzyme/protein treatment. For enzyme treatment, WT or double mutant hERp57 was added in an amount equimolar with the IZUMO1-JUNO complex. PDI storage buffer was used as a negative control. For the competitive complex positive control, wells were treated with 1 µg free unlabeled IZUMO1-JUNO complex. For the nanobody positive control, a molar ratio of 1.21 to 1 of anti-IZUMO1 nanobody ZNb11 (custom generated by Lee Lab) to bait complex was used. PBS only was used as a negative control. The plate was incubated with the treatments for 1 h at RT, followed by three washes with 200 µL of wash buffer. Finally, 100 µL of 1X PBS pH 7.4 was added to each well, and the fluorescence intensity was read on a Synergy Neo2 multimode microplate reader (BioTek/Agilent Technologies) with excitation of 487 nm, emission of 528 nm, and gain of 140.

### AlphaFold 3 protein–protein complex modeling

Computational protein modeling was used to evaluate the potential interaction between hERp57 and IZUMO1 or IZUMO1-JUNO complex. Protein–protein complex modeling was performed using the AlphaFold 3 webserver [36]. The protein sequences used in the analysis were IZUMO1 (residues 22–350), hERp57 (residues 25–505), JUNO (residues 20–250), and calnexin (residues 21–592). Known and predicted post-translational modifications were added, as allowed by the AlphaFold 3 server (Supplementary Table S2). The highest ranked model from each AlphaFold 3 prediction was selected, and pAE plots, pTM, and ipTM values were derived from these top models. pAE plots were generated using the PAE Viewer webserver [37].

### Quantification and statistical analysis

Statistical analysis was performed using GraphPad Prism (version 9.0) for Windows (GraphPad Software, La Jolla California USA). Detailed description of the statistical methods is provided in the figure legends for each experiment. Differences were considered statistically significant when the p-value was less than 0.05. Data are expressed as mean ± SEM of at least three independent experiments.

## Results

### PDI activity is required for mouse in vitro fertilization

We first wanted to understand whether PDI activity is required during gamete interaction and fertilization. Prior to performing mouse IVF studies, we characterized the effectiveness of two known chemical inhibitors on hERp57 activity: bacitracin, which is a membrane-impermeable inhibitor of global PDI activity [38, 39], and punicalagin, which was initially described as a selective inhibitor of PDIA3 (ERp57) [40] and later as an ERp57 and PDIA1 selective inhibitor [41]. We expressed wildtype (WT) hERp57 as well as a catalytically dead mutant with both thioredoxin-like active sites mutated from CXXC to AXXA (Supplementary Figure S1). hERp57 WT activity in solution was significantly inhibited with punicalagin in micromolar dose-dependent amounts (Supplementary Figure S2). In contrast, incubation with bacitracin showed minimal inhibition (Supplementary Figure S2). This indicates that punicalagin is a specific and potent inhibitor of hERp57 activity, while bacitracin is not, and is likely an inhibitor to other PDI classes.

**Fig. 1:**
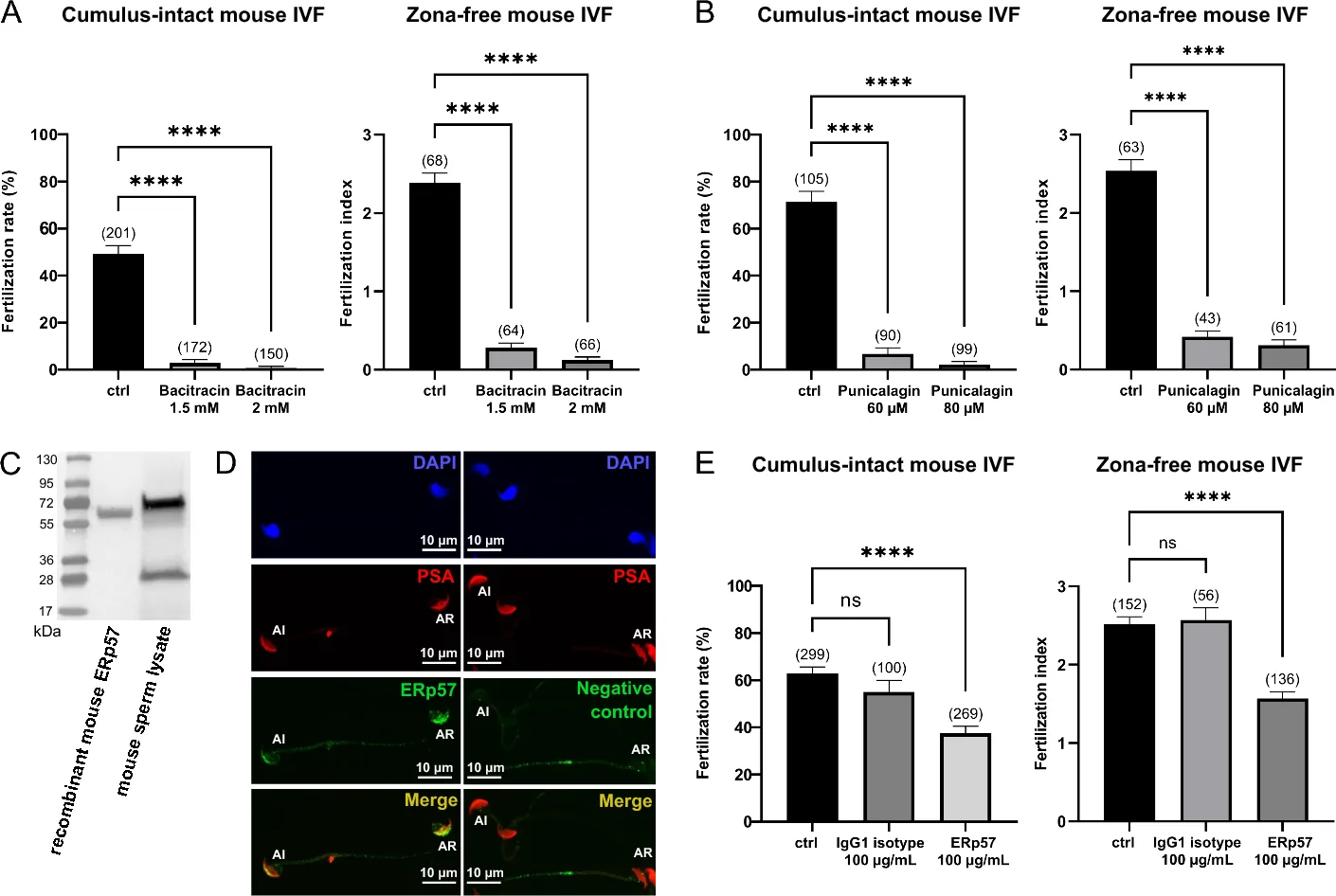
Inhibition of ERp57 and other PDIs reduces mouse fertilization in vitro. A cumulus-intact and zona-free mouse IVF assay was performed in the presence and absence of **A** bacitracin, a broad-spectrum inhibitor of PDIs, and **B** punicalagin, a selective inhibitor of PDIA3 (ERp57) and PDIA1 family. The fertilization rate and fertilization index (mean ± SEM) were measured for the cumulus-intact or zona-free IVF assays, respectively. **C** Mouse ERp57 (expected band at ~ 60 kDa) was detected using the mouse anti-hERp57 antibody (Santa Cruz Biotechnology, clone 4E69). Western blot analysis demonstrates cross-reactivity with recombinant mouse ERp57 protein, as well as with mouse sperm. **D** Immunofluorescence staining of ERp57 on acrosome intact (AI) and acrosome reacted (AR) mouse spermatozoa (green). Acrosome staining was confirmed using rhodamine-conjugated Pisum Sativum Agglutinin (PSA) (red), and nuclei were stained with DAPI (blue). **E** A cumulus-intact and zona-free mouse IVF assay was performed with a mouse anti-hERp57 antibody or IgG1 isotype control antibody. ****p < 0.0001 by an ordinary one-way ANOVA test for cumulus-intact IVF in Fig. 1D and by Brown-Forsythe and Welch one-way ANOVA tests for all other experiments presented in Fig. 1. All experiments were performed with a minimum of three biological replicates. The number of oocytes in each experiment is indicated in parentheses.

After investigating the effects of various inhibitors, cumulus-intact and zona-free mouse oocytes from WT females were inseminated with spermatozoa from WT males in the presence of bacitracin, punicalagin, or a mouse anti-hERp57 antibody. In the cumulus-intact mouse IVF assays, the inhibitors caused a drastic reduction in FR, which is defined as the average number of fertilized oocytes relative to the total number of oocytes, compared to the control (Fig. 1A and 1B). For the zona-free mouse IVF assays, a similar significant decrease in FI was observed. The FI, which represents the number of decondensed spermatozoon heads per oocyte, dropped 8.6-fold (0.28 ± 0.06) with 1.5 mM bacitracin, 20-fold (0.12 ± 0.04) with 2 mM bacitracin, sixfold (0.42 ± 0.08) with 60 μM punicalagin, and 8.1-fold (0.31 ± 0.07) with 80 μM punicalagin (Fig. 1A and 1B). These findings support the involvement of PDI activity, specifically ERp57, in fertilization.

To further assess the specific role of ERp57, we used a mouse anti-hERp57 antibody to selectively inhibit ERp57 activity. Western blot analysis confirmed that the anti-hERp57 antibody cross-reacts with mouse ERp57 from recombinant purification or spermatozoa lysate (Fig. 1C). Immunofluorescence staining revealed specific localization to the acrosomal region in acrosome intact spermatozoa and to the equatorial segment and post-acrosomal region of acrosome-reacted spermatozoa (Fig. 1D). No specific signal was observed with the IgG1 isotype control, aside from low background staining on the flagellum. IVF assays with the mouse anti-hERp57 antibody showed a significant decrease in both the FR and FI (~ 40% lower than the control). Specifically, the FR for the WT control was 62.9% compared to 37.5% with 100 μg/mL anti-hERp57 antibody. Similarly, the FI for the WT control condition was (2.5 ± 0.09) compared to (1.6 ± 0.09) with the anti-hERp57 antibody. No significant difference was found between the WT positive and the IgG1 isotype control groups (Fig. 1E). These results suggest that inhibition of surface ERp57 via antibody binding impairs in vitro fertilization in mice. However, the extent of inhibition observed with the antibody was markedly less than that caused by chemical inhibitors such as bacitracin or punicalagin. This partial effect likely reflects the mode of binding to mouse ERp57, which may not fully block ERp57 activity on mouse spermatozoa.

### *ERp57* conditional knockout mice generation and genotyping

To evaluate the role of ERp57 in spermatozoa, we generated male mice with a conditional KO of *ERp57* specifically in spermatozoa. This was accomplished by crossing *ERp57* floxed mice [28] with transgenic mice expressing Cre recombinase under the control of a 1.4 kb promoter region from the germ cell-specific *Stimulated by retinoic acid gene 8* (*Stra8*) [29]. *Stra8-cre* drives Cre recombinase expression in pre-meiotic male germ cells, including early-stage spermatogonia and pre-leptotene-stage spermatocytes. ERp57 is expressed throughout all stages of spermatogenesis, as shown by bulk transcriptomic analysis in mice [42–44], and by single cell transcriptomics in mice [45, 46] and humans [47]. As a result, the resulting scKO males should have *ERp57* deleted in most of their spermatozoa. To demonstrate the excision of the floxed allele, we performed PCR on spermatozoa DNA from homozygous floxed males, either with or without *Stra8-cre* expression. In Cre-expressing males, a specific PCR band corresponding to the deleted allele was observed (Supplementary Figure S3, crossing A), which was absent in control spermatozoa lacking Cre.

Given that *Stra8-cre* efficiency is not 100% (> 95% as reported by Sadate-Ngatchou et al*.* [29]), the excision could be optimized by using scKO males with one deleted *ERp57* allele and one floxed *ERp57* allele, along with heterozygous expression of *Stra8*-*cre* (Supplementary Figure S3, crossing B). In this case, the same amount of Cre recombinase needs to excise only a single floxed allele, thereby increasing the likelihood of complete recombination. The spermatozoa of these scKO males, although genotypically heterozygous for *cre,* are phenotypically expressing Cre since mRNAs and proteins pass from one cell to another through the cytoplasmic bridges connecting the sister cells together; spermatogenesis forms a syncytium. As controls, we used males with one *ERp57* deleted allele and one floxed *ERp57* allele, but without expression of *Stra8*-*cre*. In this case, given that half of the alleles are already deleted, both in scKO (Cre positive) and control (Cre negative) males, the band indicating the occurrence of a deletion in spermatozoa DNA is always present and does not allow for discrimination between scKO and controls. Despite this optimization and given the incomplete efficiency of Cre recombinase described in the literature [48], we cannot exclude that a small percentage of spermatozoa from scKO males escaped excision. Western blot analysis revealed that ERp57 levels were generally lower in the sperm of scKO males compared to those of control males (Supplementary Figure S4).

### ***ERp57*** scKO male mice show hypofertility in vivo and in vitro but their spermatozoa do not accumulate in the perivitelline space

**Fig. 2:**
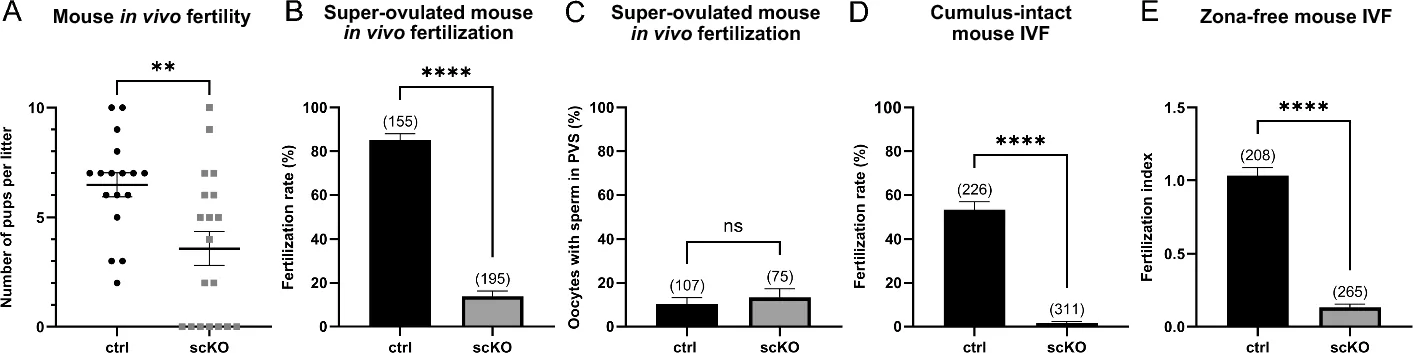
*ERp57* spermatozoa conditional knockout male mice show hypofertility in vivo and in vitro. **A** WT females were mated with control (ctrl) or scKO males, and litter size (mean ± SEM) was counted 3 weeks post-mating, confirmed by the presence of a vaginal plug. **p < 0.01 by Mann–Whitney test. **B** Superovulated WT females were mated with ctrl or scKO males to evaluate in vivo fertilization. ****p < 0.0001 by unpaired t-test. **C** The number of oocytes with spermatozoa in their PVS has been compared in both conditions and showed no significant difference (ns). p > 0.05 by unpaired t-test. **D** Cumulus-intact mouse IVF assays were performed with control or scKO sperm. ****p < 0.0001 by Welch’s t-test. **E** Zona-free mouse IVF assays were performed with control or scKO sperm. ****p < 0.0001 by Welch’s t-test. All experiments were performed with a minimum of three biological replicates. Numbers between parentheses indicate oocyte counts.

The in vivo fertility of scKO males was investigated here for the first time. Control and scKO littermate males were mated with WT females. The scKO males showed normal mating behaviors, comparable to the control males. A litter was considered as null when females exhibited a vaginal plug after mating but did not give birth to live pups. The average litter size was significantly smaller for pups born from scKO males (3.6 ± 0.8) compared to those born from control males (6.5 ± 0.5) (Fig. 2A). In vivo fertilization assays were conducted with control or *ERp57* scKO males mated overnight with superovulated WT females. The following day, females showing a vaginal plug were sacrificed, and cumulus-intact oocytes were retrieved to assess the in vivo FR. The FR was significantly lower in the scKO condition (13.8%) compared to controls (85.2%) (Fig. 2B).

In vitro fertilization assays were then performed with cumulus-intact and zona-free mouse oocytes from WT females, incubated with spermatozoa from control or scKO males. Spermatozoa from the scKO mice showed comparable count, motility, and morphology to spermatozoa from the control mice, which is notably different from the abnormal spermatozoa observed in the P5 conditional KO mice [26]. For cumulus-intact IVF assays, fertilization was severely reduced in the scKO condition (1.6%) compared to the control (53.5%) (Fig. 2D). In zona-free IVF assays, the FI for scKO males was (0.13 ± 0.02), whereas it was (1.03 ± 0.05) for controls (Fig. 2E). These observations suggest that the deletion of *ERp57* in spermatozoa severely impairs mouse fertilization both in vivo and in vitro (Supplementary Figure S5). The inhibitory effect of *ERp57* deletion on fertilization is more profound in vitro than in vivo.

To better define the stage at which ERp57 deficiency leads to severe in vivo and in vitro subfertility observed in scKO males, and to assess potential similarities with the phenotypes of previously reported *Izumo1* KO males, we mated control or scKO males with superovulated WT females and recovered embryos the following day after confirming vaginal plugs. Despite a significant reduction in fertilization rate with *ERp57* scKO spermatozoa (Fig. 2B), there was no corresponding increase in spermatozoa accumulation within the PVS. Spermatozoa were detected in the PVS of only 10.3% of oocytes inseminated with WT spermatozoa and 13.3% with scKO spermatozoa (Fig. 2C). This contrasts sharply with findings in *Izumo1* KO mice [8], where spermatozoa accumulation in the PVS is a prominent feature.

### ERp57 co-localizes with IZUMO1 on the equatorial region of human spermatozoa

**Fig. 3:**
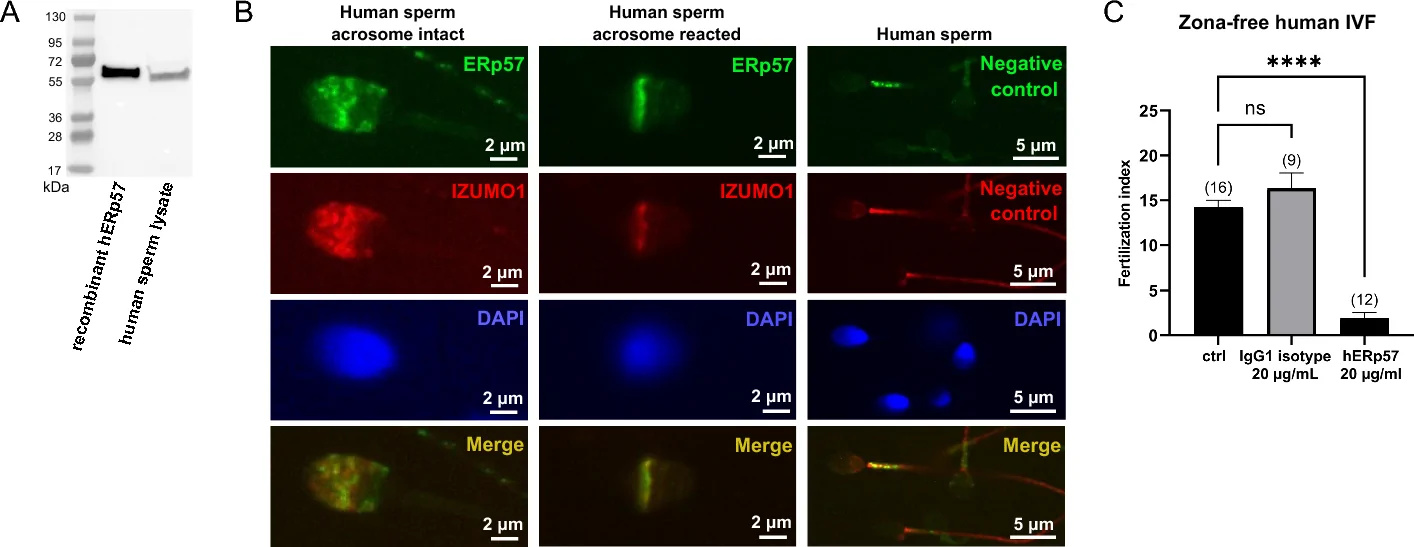
hERp57 localizes with IZUMO1 and hERp57 inhibition leads to reduced fertilization in human zona-free IVF. **A** Human ERp57 (expected band at ~ 60 kDa) was detected using the mouse anti-hERp57 antibody (Santa Cruz Biotechnology, clone 4E69). Western blot analysis demonstrates cross-reactivity with recombinant human ERp57 protein, as well as with human sperm. **B** Immunofluorescence staining of permeabilized human acrosome-intact and acrosome-reacted spermatozoa revealed that hERp57 (in green) and IZUMO1 (in red) relocate from the acrosome cap of acrosome-intact spermatozoa to co-localize at the equatorial segment following the acrosome reaction (yellow in merged images). No non-specific staining was observed on the spermatozoon head in the IgG1 isotype negative control, although background staining appeared on the flagellum. Nuclei were stained with DAPI (in blue). **C** Zona-free human IVF assay was performed with or without a mouse anti-hERp57 antibody and an IgG1 isotype control. The number of decondensed spermatozoa heads per zona-free oocyte was recorded to assess the fertilization index (mean ± SEM). ns: no significant difference, ****p < 0.0001 by Kruskal–Wallis one-way ANOVA test. All experiments included a minimum of three biological replicates. Numbers between parentheses indicate the number of oocytes.

We next wanted to understand whether hERp57 is displayed on the surface of human spermatozoa and co-localizes with IZUMO1. As in mice, Western blot analysis confirmed that the anti-hERp57 antibody cross-reacts with human ERp57 from recombinant purification or spermatozoa lysate (Fig. 1C; Fig. 3A). Also similar to mice, we observed a specific acrosomal punctiform staining on acrosome-intact human spermatozoa and staining at the equatorial segment in the post-acrosomal region of acrosome-reacted human spermatozoa heads (Fig. 1D; Fig. 3B). No signal, apart from background noise present on the flagellum, was observed with the IgG1 isotype control antibody. This relocation of hERp57 from the acrosome to the equatorial segment is similar to localization of IZUMO1 after the acrosome reaction (Fig. 3B). This result is consistent with a role for hERp57 in gamete interaction and potentially in the thiol-disulfide exchange reaction of IZUMO1.

### Anti-hERp57 antibody has an inhibitory effect on human in vitro fertilization

Zona-free human IVF assays were performed in the presence of the mouse anti-hERp57 or mouse IgG1 isotype control antibodies (negative control), both at a concentration of 20 µg/mL. Spermatozoa were pre-incubated with either the anti-hERp57 antibody or an anti-IgG1 isotype control antibody prior to insemination. The FI significantly decreased from 14.3 for the control group to 1.9 for the anti-hERp57 treated group, representing an 87% reduction of fertilization efficiency (Fig. 3C). No significant difference was observed between the IgG1 isotype control (FI = 16.3) and the control group, which received no antibody treatment. These results suggest that inhibition of ERp57 disrupts human in vitro fertilization.

### IZUMO1 displays a pair of surface-exposed CXXC motifs

**Fig. 4:**
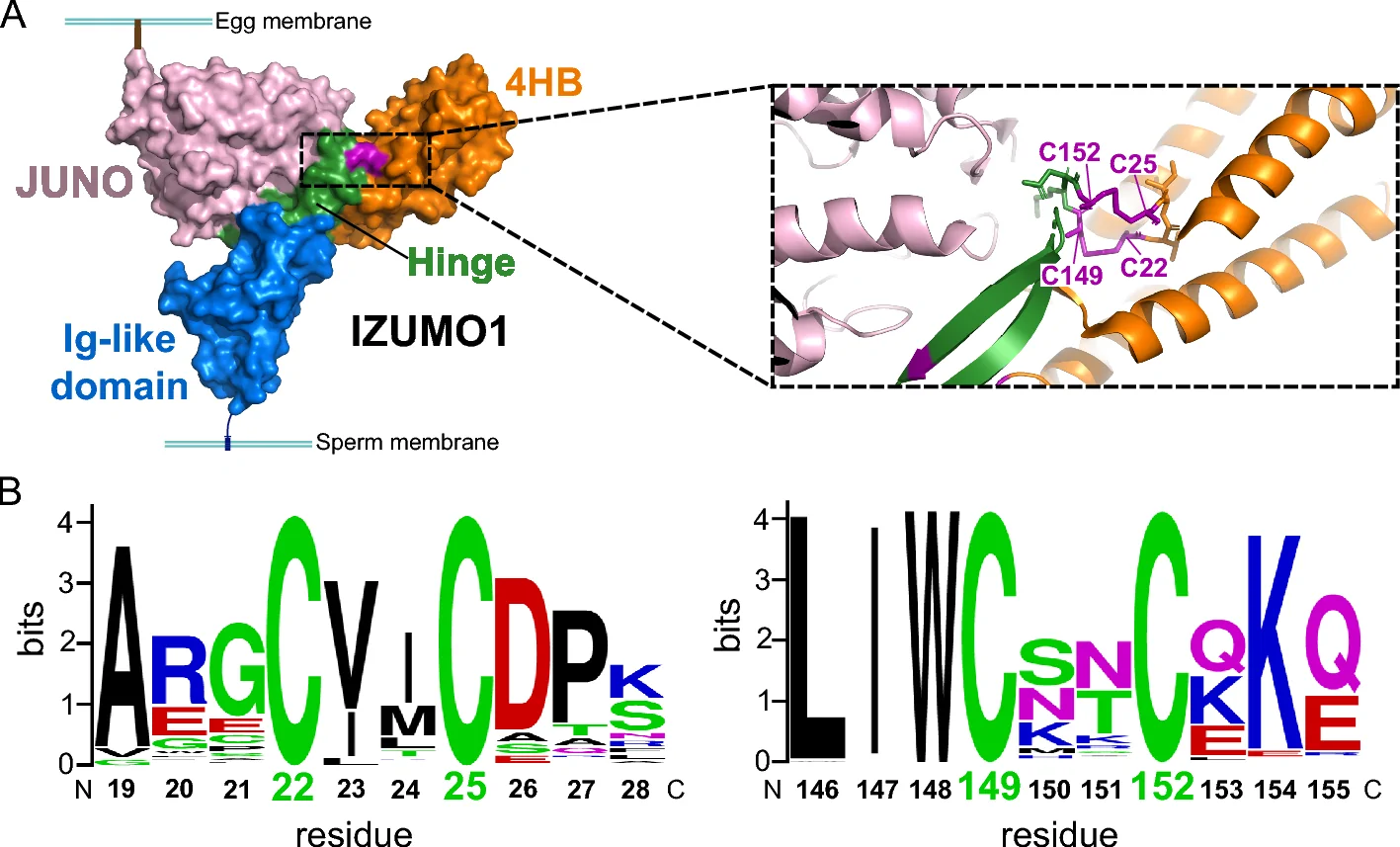
IZUMO1 contains a pair of conserved surface-exposed disulfide-bonded CXXC motifs. **A** A surface model of the IZUMO1-JUNO complex is displayed, with a zoomed-in view highlighting the surface-exposed disulfide-bonded CXXC motifs in IZUMO1. JUNO is shown in red and IZUMO1 is colored by domain: four-helix bundle (4HB) in orange, immunoglobulin (Ig)-like domain in blue, and hinge region in green (PDB: 5F4E). Cysteines are highlighted in purple. Representations of the spermatozoa and oocyte membranes are included for context. **B** A multiple sequence alignment of 88 mammalian IZUMO1 sequences from UniProt [32] (accession numbers listed in Supplementary Table S1) was performed using Clustal Omega [33, 34], and logo plots were generated by WebLogo (35). Residue numbers listed correspond to the human IZUMO1 sequence.

Several structures of human IZUMO1 and IZUMO1-JUNO complexes were previously determined [5, 7, 49] and are in full agreement. A deeper analysis of the IZUMO1-JUNO structure revealed an interesting pair of surface-exposed CXXC motifs in the 4-helix bundle (4HB) domain (Fig. 4A) that have not been described in previous literature. CXXC motifs are commonly found in thioredoxin-like enzymes and can act as redox sensors [50, 51]. In the case of IZUMO1, the ^22^C-X-X-C^25^ and ^149^C-X-X-C^152^ motifs are not reduced, as commonly observed in thioredoxin-like enzymes, but instead the cysteines form inter-motif disulfide bonds (Fig. 4A). Cys22 forms a disulfide linkage with Cys149, while Cys25 forms a second disulfide to Cys152. As a result, these two disulfide bonds form a covalent link between the 4HB and the hinge region. Both CXXC motifs are solvent accessible on the surface in the IZUMO1-JUNO complex and unbound IZUMO1 (Fig. 4A). Moreover, the pair of CXXC motifs is conserved in all mammalian IZUMO1 proteins (Fig. 4B). Altogether, this led us to question whether the surface-exposed CXXC motifs are the targets of hERp57 to trigger dimerization in IZUMO1, thus leading to the downstream steps of sperm-egg fusion.

### Reduction of IZUMO1 disulfide bonds leads to oligomerization

**Fig. 5:**
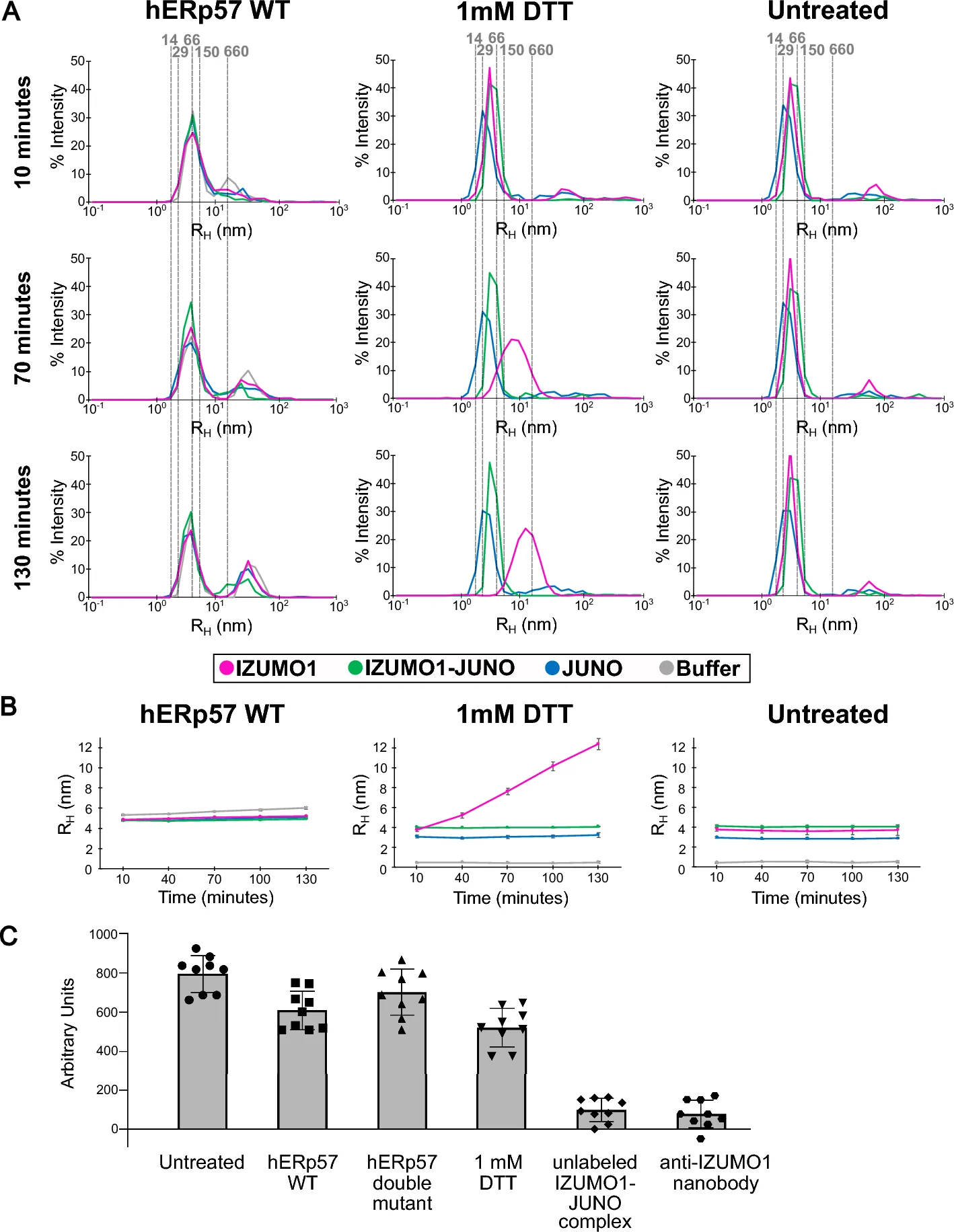
hERp57 treatment in solution does not lead to IZUMO1 dimerization or dissociation from JUNO. Dynamic light scattering (DLS) analysis was used to assess oligomerization of IZUMO1 in solution. **A** The percent intensity peaks are shown, with the average hydrodynamic radius (R_H_) of the protein standard markers indicated by dashed lines. Numbers above the dashed lines refer to the protein standard molecular weight in kDa. Oligomerization occurs only when IZUMO1 is incubated with dithiothreitol (DTT), not with hERp57 WT. **B** The R_H_ over time confirms the absence of IZUMO1 oligomerization with hERp57 WT. **C** A biotinylated IZUMO1-JUNO complex labeled with FITC on JUNO was treated with hERp57 WT, hERp57 catalytically dead double mutant, unlabeled IZUMO1-JUNO complex, an anti-IZUMO1 nanobody, or no treatment. A decrease in fluorescence would indicate dissociation of the labeled complex. All data is baseline subtracted with unlabeled IZUMO1-JUNO complex. Three technical replicates from three independent replicates were performed for each sample.

To determine whether IZUMO1 can oligomerize, we developed a real-time dynamic light scattering (DLS)-based assay to quantify the particle size distribution in solution over time. DLS tracks the scattering of polarized light by particles and uses the autocorrelation function to determine the diffusion coefficient, from which particle size (hydrodynamic radius, R_H_) is calculated. This DLS assay has the advantage of being able to quantitate and monitor the formation of oligomers in real-time. We first validated the assay with known protein standards, demonstrating a molecular weight-dependent increase in light scattering, supporting its use for detecting IZUMO1 oligomerization (Supplementary Figure S6). Using this assay, we asked the question whether IZUMO1 can oligomerize if the disulfides are disrupted. DTT, a strong reducing agent, has been previously shown by Ohto et al*.* to trigger IZUMO1 oligomerization [7]. Consistent with this, we incubated IZUMO1 alone or in complex with JUNO (Supplementary Figure S7) in the presence of 1 mM DTT and monitored R_H_ over a 2 h period. A significant increase in particle size from 4 to 12 nm was observed for IZUMO1 alone, indicating oligomer formation (Fig. 5). In contrast, no change in R_H_ was detected when IZUMO1 was complexed with JUNO. No protein precipitation was observed in any samples. The DLS measurements were consistent with the reported dimensions of IZUMO1 (~ 8.5 × 2.4 × 2.0 nm) and JUNO (~ 3.4 × 2.5 × 2.1 nm).

### hERp57 does not modify or trigger IZUMO1 oligomerization

We used our DLS assay described above to test if IZUMO1 oligomerizes in the presence of recombinant hERp57. IZUMO1, JUNO, and IZUMO1-JUNO complex were incubated with equimolar hERp57 WT or catalytically dead double mutant and the R_H_ of each of these samples was measured over a 2 h period. No relevant change in R_H_ was observed in any sample, indicating a lack of IZUMO1 oligomerization (Fig. 5AB). The addition of hERp57 WT or double mutant led to a double peak, including a higher molecular weight species; however, this peak was also present with hERp57 when only JUNO or buffer was added, showing that this higher molecular weight species is not IZUMO1 oligomer.

In order to confirm that hERp57 WT does not reduce the disulfide bonds in IZUMO1, we incubated IZUMO1 and hERp57 WT with maleimide, which specifically conjugates to free thiol groups, and analyzed the samples using electrospray ionization mass spectrometry. While hERp57 WT showed peaks indicating maleimide modification on its catalytic cysteines, IZUMO1 showed no maleimide addition (Supplementary Figure S8), confirming that hERp57 does not reduce or modify the disulfide bonds in IZUMO1.

### hERp57 does not trigger dissociation of the IZUMO1-JUNO complex

We also investigated whether hERp57 triggers dissociation of the IZUMO1-JUNO complex, which is theorized to enable IZUMO1 oligomerization. We complexed biotinylated IZUMO1 with fluorescein isothiocyanate (FITC)-labeled JUNO and added this complex to a streptavidin-coated plate, followed by hERp57 treatment. Dissociation was measured by a decrease in fluorescence, due to the loss of JUNO-FITC. However, hERp57 did not induce significant JUNO dissociation, as fluorescence values for hERp57 WT treatment (610 ± 33 a.u.) were similar to those for the catalytically inactive hERp57 double mutant (703 ± 39 a.u.) and the untreated control (795 ± 31 a.u.) (Fig. 5C). In contrast, positive control treatments, including an unlabeled IZUMO1-JUNO complex or an anti-IZUMO1 nanobody that inhibits IZUMO1-JUNO complex formation, showed large decreases in fluorescence values (99 ± 20 a.u.) and (79 ± 24 a.u.), respectively (Fig. 5C). These results suggest that hERp57 does not trigger dissociation of JUNO from the IZUMO1-JUNO complex.

### IZUMO1 is not predicted to interact with hERp57

AlphaFold 3 modeling predicted no interaction between hERp57 and IZUMO1, either alone or in complex with JUNO. The interface predicted template modeling (ipTM) scores for these interactions are 0.26 (IZUMO1-hERp57) and 0.48 (IZUMO1-JUNO-hERp57), both below the suggested 0.6 threshold for a successfully predicted interaction (Supplementary Figure S9). These models also showed high predicted aligned error (pAE) between IZUMO1 and hERp57. In contrast, the predicted interaction between hERp57 and calnexin, a natural hERp57 substrate [52, 53], has a higher ipTM value of 0.68, indicating moderate predicted interaction confidence (Supplementary Figure S9). While the pAE plot for calnexin-ERp57 has regions of high error, regions of high confidence reside around calnexin_300-380_ and hERp57_135-345_, suggesting a possible site of interaction (Supplementary Figure S9). As a control, AlphaFold 3 was able to predict the known IZUMO1-JUNO binding pair (ipTM value of 0.79 and little error), confirming the high confidence of this known interaction. Overall, while known interactions (hERp57-calnexin and IZUMO1-JUNO) were supported by the AlphaFold 3 modeling, hERp57 was not predicted to interact with IZUMO1 or IZUMO1-JUNO, consistent with our experimental findings.

## Discussion

In this study, we investigated the role of ERp57 in fertilization using a combination of genetic, biochemical, and computational approaches. We first applied biochemical and in vitro strategies to prove ERp57 involvement in fertilization. The fact that bacitracin, which is not specific to ERp57, inhibited fertilization as efficiently as punicalagin suggests that other spermatozoa and/or oocyte PDIs could be involved in gamete adhesion/fusion. Fertilization inhibition by an anti-hERp57 antibody was significantly more effective in humans compared to mouse, likely because the antibody was initially raised against recombinant human ERp57.

Mouse KO studies are a crucial tool for determining the roles of various proteins within complex mechanisms, including fertilization. These models are particularly valuable in reproductive biology to determine if a given protein is critical for sperm-egg membrane fusion, ZP penetration, or other stages of fertilization. Since global *ERp57* KO is embryonically lethal [28], we created a conditional KO mouse model targeting *ERp57* deletion specifically in spermatozoa. This was achieved by crossing a floxed *ERp57* mouse line with one expressing Cre recombinase in premeiotic male germ cells. Previous studies suggested that ERp57 localized on the spermatozoon equatorial region is important for fertilization, with antisera against ERp57 reducing fertilization rates [25]. Additionally, blocking sulfhydryl groups on spermatozoa proteins also inhibits fertilization [54, 55], indicating a role for PDIs. Our results clearly reveal the direct involvement of PDI activity in fertilization, as chemical inhibitors, specific antibodies, and *ERp57* scKO mice resulted in significantly reduced fertilization both in vivo and in vitro, though more drastically in vitro. This difference is most likely due to the distinction between optimal in vivo fertilization and the artificial context of IVF where some parameters are not ideally reproduced. Thus, IVF could exacerbate a defect that is otherwise compensated for under physiological conditions. This difference of phenotype between in vivo and in vitro conditions has already been observed for different KOs [56–58]. In addition, the phenotypic variability between scKO males regarding in vivo fertility is most likely due to variation in the efficiency of floxed site excision by Cre recombinase between different males and different waves of spermatogenesis for the same male. This variability was also observed in Western blot analyses showing different residual levels of ERp57 in spermatozoa from different scKO males (Supplementary Figure S4). Furthermore, the role of ERp57 complements those of other members of the PDI family, such as ERp29 [59] and PDILT [60] in reproduction. The requirement of PDIs in fertilization has also been observed in parasites like *Plasmodium berghei* and *Neospora caninum,* where PDI-Trans and NcPDI, respectively, are essential for fertilization [61, 62].

The localization of ERp57 on the spermatozoon membrane is intriguing since PDIs are typically found in the ER. It is unknown how ERp57 escapes the ER, especially considering that it lacks a transmembrane domain of its own. It is likely that ERp57 interacts with other membrane-associated proteins to escape the ER and retain its positioning at the spermatozoon membrane surface. Localization of ERp57 specifically to the equatorial segment of spermatozoa in both mice and humans is particularly noteworthy, as this region serves as the primary site for sperm-egg adhesion. This area also displays several key fertilization factors, including IZUMO1, SPACA6, TMEM81, TMEM95, FIMP, DCST1 and DCST2. Notably, this spatial distribution aligns with the model proposed by Inoue et al., which suggests that following the binding of IZUMO1 to JUNO, a disulfide isomerase catalyzes IZUMO1 dimerization into a closed conformation and facilitates JUNO dissociation [20]. Interestingly, structural analysis of IZUMO1 reveals four highly conserved cysteine residues arranged in two CXXC motifs that form intramolecular disulfide bonds. These motifs remain surface exposed even when IZUMO1 is bound to JUNO. Furthermore, chemical reduction of IZUMO1 induces oligomerization, suggesting that redox regulation of the CXXC motifs may promote oligomeric states of IZUMO1.

Thioredoxin-like CXXC motifs are well-known redox sensors, which can undergo structural rearrangements in response to changes in the redox environment [50, 51]. CXXC motifs and PDIs have been implicated in membrane fusion processes across various viruses. For instance, in human T-cell leukemia virus-1 (HTLV-1), the attachment subunit (SU) of its class I viral glycoprotein fusogen is disulfide linked to its fusion subunit (TM). Isomerization of the disulfide bond by activating a thiol group in the CXXC motif in SU results in the dissociation of the attachment subunit, triggering fusion [63]. Similar mechanisms have also been observed in murine leukemia virus (MLV) [64, 65]. In Sindbis virus, its viral envelope glycoprotein (E) is stabilized by disulfide bonds and cell surface reduction of these disulfides leads to disruption of the rigid protein–protein associations to trigger fusion [66]. In the case of hepatitis B virus (HBV), ERp57 is crucial for membrane fusion to human cells [67], but its underlying mechanisms are still unknown. These examples have led to the prominent hypothesis within the reproductive biology field that ERp57 could act as the trigger for IZUMO1 dimerization. While we show IZUMO1 is capable of oligomerizing when its disulfide bonds are chemically reduced, our biophysical and computational data showed no evidence of an interaction between ERp57 and IZUMO1, either alone or in complex with JUNO. Moreover, hERp57 does not induce IZUMO1 dimerization. Further evidence against a direct role for ERp57 in sperm-egg fusion comes from our observation that ERp57-deficient spermatozoa do not accumulate in the PVS following in vivo fertilization. This contrasts with IZUMO1 or SPACA6 KO mouse models, in which spermatozoa penetrate the ZP but fail to adhere to or fuse with the oolemma, resulting in PVS accumulation. Collectively, these findings suggest that ERp57 functions upstream of membrane fusion, likely by modulating earlier stages of fertilization. For instance, one study implicated that spermatozoon surface ERp57 enhances ZP-binding capacity following capacitation through increased surface thiol content [68]. In future studies, kinetic substrate trapping could be used to identify target molecules for ERp57 [69–71], although this approach has several limitations. Generating a stable cell line expressing an ERp57 kinetic trap mutant is not feasible in human gametes, and the addition of an exogenous ERp57 mutant would both limit detection to extracellular targets and require large quantities of gametes.

## Conclusions

Despite insights such as JUNO shedding from the sperm-egg fusion interface [19] and IZUMO1 dimerization in vitro [20], the molecular events following initial IZUMO1-JUNO binding remain largely unresolved. It is now evident that ERp57 does not trigger IZUMO1 dimerization nor play a direct role in the fusion of spermatozoon and oocyte membranes. This raises a key question of what triggers IZUMO1 multimerization? The observation that DTT reduction of IZUMO1 disulfides leads to oligomerization suggests that multimerization could be triggered via disruption of its disulfides. The presence of CXXC motifs on IZUMO1 raises the possibility that these sites are targeted by an as-yet unidentified activation factor. Intriguingly, SPACA6, and TMEM95 − all members of the IST superfamily along with IZUMO1 [72] − are also key fertilization factors that localize to the equatorial segment, and they too feature similar surface-exposed CXXC motifs. Recent experimental studies suggest IZUMO1 is part of a larger pre-fertilization spermatozoon complex involving human IZUMO1-SPACA6-TMEM81 [13, 73]. Structural modeling of this complex reveals several surface-exposed, disulfide-bonded cysteine residues, including CXXC or CX_n_C motifs (Supplementary Figure S10). The possibility remains that reduction or isomerization of IZUMO1 or associated complexes may occur at the sperm-egg fusion interface through other PDIs, reductases or redox mechanisms. Furthermore, the activation factor needs not be confined to the spermatozoon surface but could be present on the oocyte membrane. Further research into the roles of the CXXC motif and IZUMO1 activation factors − both on the spermatozoon and oocyte plasma membranes − will be critical for elucidating the mechanisms underlying gamete fusion. A deeper understanding of this process could pave the way for improved infertility treatments and the development of non-hormonal contraceptive methods. We present these findings to encourage a re-examination of current models of sperm-egg fusion.

## Supplementary Information

Additional file 1: This is the main file that contains the Supplementary Figures and Tables. Supplementary Figure S1: Validation of recombinant hERp57 WT and hERp57 double mutant; Supplementary Figure S2: Inhibition of hERp57 WT by bacitracin and punicalagin; Supplementary Figure S3: Generation and genotyping of a conditional knockout of *ERp57* in sperm; Supplementary Figure S4: Western blots show lower ERp57 expression levels in scKO male mice compared to controls. Supplementary Figure S5: Individual *ERp57* scKO mice fertility data supports hypofertility; Supplementary Figure S6: Dynamic Light Scattering (DLS) protein standard curve; Supplementary Figure S7: Validation of recombinant IZUMO1, JUNO and IZUMO1-JUNO complex; Supplementary Figure S8: Lack of disulfide bond reduction in IZUMO1 when incubated with hERp57 WT; Supplementary Figure S9: hERp57 and IZUMO1 are not predicted to directly interact; Supplementary Figure S10: Predicted human IZUMO1-SPACA6-TMEM81 complex displays many surface-exposed cysteine residues; Supplementary Table S1: IZUMO1 Uniprot sequence identifiers and organism names used for multiple sequence alignment; Supplementary Table S2: Known and predicted post-translational modifications of IZUMO1, hERp57, JUNO and calnexin used for AlphaFold 3 predictions.

Additional file 2: Full Western Blot & Gel Images (pdf): The full Western blot and gel images, and their replicates, as appropriate, relating to Fig. 1C, Fig. 3A, Supplementary Figure S1B, Supplementary Figure S3, Supplementary Figure S4, and Supplementary Figure S7A are included. Each image shows a red dashed-line box which highlights the portion of each image that was used in the corresponding figure.

## Data Availability

All data reported in this paper will be shared by the lead contacts upon request.

## Abbreviations

COC: Cumulus oocyte complex
DLS: Dynamic light scattering
ER: Endoplasmic reticulum
FI: Fertilization index
FITC: Fluorescein isothiocyanate
FR: Fertilization rate
hCG: Human chorionic gonadotropin
ipTM: Interface predicted template modeling
IVF: In vitro fertilization
KO: Knock-out
pAE: Predicted aligned error
PDI: Protein disulfide isomerase
PMSG: Pregnant mare serum gonadotropin
pTM: Predicted template modeling
PVS: Perivitelline space
R_H_: Hydrodynamic radius
scKO: Spermatozoa conditional knock-out
SEC: Size exclusion chromatography
*Stra8*: *Stimulated by retinoic acid gene 8*
ZP: Zona pellucida
