# Supplementary material for: Sperm surface protein disulfide isomerase ERp57 is crucial for mammalian fertilization but functions independently of IZUMO1
Source: Cell Mol Biol Lett. 2026 Jun 27;31:149. doi: 10.1186/s11658-026-00984-y (PMC13599313; doi:10.1186/s11658-026-00984-y)
Supplement: Supplementary file 1 [file 11658_2026_984_MOESM1_ESM.docx]

**MAIN SUPPLEMENTAL INFORMATION**

**Figure S1. Validation of recombinant hERp57 WT and hERp57 double mutant.** (A) Electrospray ionization mass spectrometry (ESI-MS) spectra of recombinant hERp57 WT and hER57 double mutant. The ESI-MS confirms the expected molecular weights for the recombinant enzymes. (B) Coomassie-stained SDS-PAGE and Western blot analysis of recombinant hERp57 WT, hERp57 double mutant, and commercial recombinant hERp57 (Abcam, ab92937). (C) Insulin turbidity assay of hERp57 WT, commercial hERp57 and hERp57 double mutant. The hERp57 WT produced in our lab has better activity than the commercial product. All data were reference buffer subtracted.

**Figure S2. Inhibition of hERp57 WT by bacitracin and punicalagin.** Insulin turbidity assay of recombinant hERp57 treated with various concentrations of punicalagin and bacitracin. Turbidity was measured by absorbance (A_650_) over 60 minutes. Inset bar graph shows the percent hERp57 activity at the 40-minute time point. All data are reference buffer subtracted.

**Figure S3. Generation and genotyping of a conditional knockout of *ERp57* in spermatozoa.** scKO males were generated through a series of crosses. Homozygous floxed *ERp57* mice and homozygous *Stra8*-*cre* mice were crossed to produce double heterozygous offspring. From this stage, two alternative scenarios are possible with several possible male and female genotypes being obtained at each cross. In this figure, only the genotypes and sexes of interest are shown, other genotypes are symbolized by (…). The top right inset describes the schematic organization of the *ERp57* mouse gene, the position of LoxP sites and of primers used for genotyping somatic and spermatozoa DNA. The simplified schemes are used to detail successive crosses. For each genotype category, one representative PCR result is shown. Up1-R1 detects the floxed allele (about 700 bp for the WT *ERp57* allele and about 1000 bp for the floxed *ERp57* allele), F1-R2 detects the deleted allele (~850 bp) and Up6-Dn6 detects the remaining sequence between the two LoxP sites. Primers F3 and R3 were used to detect the presence of *Stra8-cre* (236 bp). In the first scenario (A), the obtained females (*ERp57 WT/fl Stra8-cre (+/-)*) were crossed with homozygous males (*ERp57 fl/fl Stra8-cre (-/-)*) resulting in several genotypes, two of particular interest: scKO males (*ERp57 fl/fl Stra8-cre (+/-)*) and their littermate male controls (*ERp57 fl/fl Stra8-cre (-/-)*). These double-floxed males expressing Cre in the male germline allow the production of a majority of excised spermatozoa, except those that escaped the action of Cre. Spermatozoon DNA from controls only exhibits floxed alleles due to the absence of Cre. In the top gel of the bottom panel, a semi-quantitative PCR shows the remaining floxed allele in scKO spermatozoa appeared weakly, only at 25 cycles (25x), while it appeared as soon as 20 cycles (20x) and was intense at 25 cycles for the control. Inversely, in the bottom gel of the bottom panel, amplification of the deletion band appeared only in the scKO spermatozoa DNA (left) and an internal amplification (35 cycles) confirmed the presence of the floxed allele in the control and of residual floxed allele in the scKO (right). In the second scenario (B), scKO males (*ERp57 fl/del Stra8-cre (+/-)*) with one deleted *ERp57* allele and one floxed *ERp57* allele, along with heterozygous expression of *Stra8-cre*, were generated in order to optimize the excision of floxed alleles. These scKO males (*ERp57 fl/del Stra8-cre (+/-)*) and their control littermate males (*ERp57 fl/del Stra8-cre (-/-)* could be generated by the crossing of double heterozygous males expressing Cre in their germline (*ERp57 WT/fl Stra8-cre (+/-)*) with homozygous floxed females *(ERp57 fl/fl Stra8-cre (-/-)*). At the spermatozoon level, scKO males should present a high level of excised allele while controls present half floxed alleles and half deleted ones. The gels show the presence of Cre under the control of the *Stra8* promoters and the presence of the LoxP sites on one or two alleles of ERp57 gene.

**Figure S4. Western blots show lower ERp57 expression levels in scKO male mice compared to controls.** Extracts from 2 × 10^6^ spermatozoa from 3 different scKO males were run in parallel with 2 × 10^6^ spermatozoa from control males. Mouse ERp57 (expected band at ~60 kDa) was detected using the mouse anti-hERp57 antibody (Santa Cruz Biotechnology, clone 4E69). ERp57 expression appears variable between scKO males but is consistently lower than in controls.

**Figure S5. Individual *ERp57* scKO mice fertility data supports hypofertility.** (A) *In vivo* fertility of *ERp57* scKO males by counting the number of pups per litter following mating with WT females. (B) *In vivo* fertilization rate (FR) of *ERp57* scKO males by calculating the FR after mating with WT super-ovulated females. (C) *In vitro* fertilization assays of *ERp57* scKO male spermatozoa and WT cumulus-intact oocytes. (D) *In vitro* fertilization assays of *ERp57* scKO male spermatozoa and WT zona-free oocytes.

**Figure S6. Dynamic Light Scattering (DLS) protein standard curve.** DLS measurements were performed on five standard proteins with varying molecular weights: lysozyme (14 kDa), carbonic anhydrase (29 kDa), bovine serum albumin (66 kDa), alcohol dehydrogenase (150 kDa), and thyroglobulin (660 kDa). The first panel (left) displays the hydrodynamic radius (R_H_), showing a clear increase in R_H_ with increasing molecular weight. The right panel displays the R_H_ by % intensity, further confirming the direct relationship between R_H_ peaks and molecular weight. Dashed lines indicate the molecular weight of each protein species.

**Figure S7. Validation of recombinant IZUMO1, JUNO, and IZUMO1-JUNO complex.** (A) Coomassie stained SDS-PAGE analysis of purified recombinant human IZUMO1, JUNO, and the IZUMO1-JUNO complex. (B) ESI-MS spectra of purified human IZUMO1 and JUNO.

**Figure S8. Lack of disulfide bond reduction in IZUMO1 when incubated with hERp57 WT.** IZUMO1, either alone or co-incubated with hERp57 WT, was reacted with maleimide to detect disulfide bond reduction. The reduction of a disulfide bond will lead to the addition of two maleimide molecules resulting in an approximate 194 Da mass increase. (A) Schematic of the thiol-maleimide conjugation reaction. (B) ESI-MS detection of maleimide addition to human IZUMO1 treated with hERp57. The inset boxes show no maleimide addition for IZUMO1, indicating that its cysteines remain as disulfide bonds. In contrast, hERp57 WT displays 0, 2, and 4 maleimide additions, reflecting the expected modification of its reduced catalytic cysteine residues. (C) Control reactions confirm the findings in panel B. IZUMO1 alone, when reacted with maleimide, shows no maleimide additions, consistent with fully oxidized disulfide bonds (left panel). hERp57 WT alone, when reacted with maleimide, shows 0, 2, and 4 maleimide additions, consistent with partial modifications of its catalytic cysteines (right panel).

**Figure S9. hERp57 and IZUMO1 are not predicted to directly interact.** AlphaFold 3 predictions for the specified protein-enzyme groups are shown. From top to bottom are the predicted structural models, schematic models, predicted aligned error (pAE) plots, interaction predicted template modeling (ipTM) and pTM values, and a binary interaction assessment. IZUMO1 is shown in cyan, hERp57 in magenta, JUNO in red, and calnexin in orange. Cysteine residues are highlighted in green, with arrows marking the surface-exposed CXXC motifs in IZUMO1, the CXXC active sites in hERp57, and the likely interacting cysteine pair in calnexin. The a and a’ domains of ERp57 are surrounded with a circular dashed gray outline. Full-length sequences were used for each protein, including transmembrane domains when applicable, but lacking the signal peptide sequences.

**Figure S10. Predicted human IZUMO1-SPACA6-TMEM81 complex displays many surface-exposed cysteine residues.** AlphaFold 3 prediction of the human IZUMO1-SPACA6-TMEM81 complex with transmembrane domains is displayed. IZUMO1, SPACA6, and TMEM81 are shown as molecular surfaces colored in cyan, magenta, and yellow-orange, respectively. Cysteine residues are highlighted in green. Each dashed-line box corresponds to a zoomed-in cartoon representation of this predicted structure, highlighting surface-exposed disulfide-bonded cysteine residues of each protein when in the IZUMO1-SPACA6-TMEM81 complex.

**Table S1. IZUMO1 Uniprot sequence identifiers and organism names used for multiple sequence alignment.** Related to Figure 4.

| **Uniprot Identifier** | **Organism** |
| --- | --- |
| A0A6J1XMY2\|A0A6J1XMY2_ACIJB | *Acinonyx jubatus* |
| G1M882\|G1M882_AILME | *Ailuropoda melanoleuca* |
| A0A2K5DEP7\|A0A2K5DEP7_AOTNA | *Aotus nancymaae* |
| A0A075IPE3\|A0A075IPE3_ATEGE | *Ateles geoffroyi* |
| A0A383YZK9\|A0A383YZK9_BALAS | *Balaenoptera acutorostrata scammoni* |
| A0A8B8VZ50\|A0A8B8VZ50_BALMU | *Balaenoptera musculus* |
| A0A6P5DD34\|A0A6P5DD34_BOSIN | *Bos indicus* |
| A0A4W2ILL7\|A0A4W2ILL7_BOBOX | *Bos indicus x Bos taurus* |
| A0A8C0AH79\|A0A8C0AH79_BOSMU | *Bos mutus grunniens* |
| E1BDA8\|E1BDA8_BOVIN | *Bos taurus* |
| F7H859\|F7H859_CALJA | *Callithrix jacchus* |
| A0A3Q7MFG9\|A0A3Q7MFG9_CALUR | *Callorhinus ursinus* |
| A0A9W3FTY5\|A0A9W3FTY5_CAMBA | *Camelus bactrianus* |
| A0A8B8TGY9\|A0A8B8TGY9_CAMFR | *Camelus ferus* |
| A0A8I3MBM0\|A0A8I3MBM0_CANLF | *Canis lupus familiaris* |
| C6ZEA3\|C6ZEA3_CAPHI | *Capra hircus* |
| A0A3Q0DGU8\|A0A3Q0DGU8_CARSF | *Carlito syrichta* |
| A0A8B7U7A9\|A0A8B7U7A9_CASCN | *Castor canadensis* |
| A0A8C3VXG0\|A0A8C3VXG0_9CETA | *Catagonus wagneri* |
| A0A2K5MSM3\|A0A2K5MSM3_CERAT | *Cercocebus atys* |
| A0A0D9S2Z4\|A0A0D9S2Z4_CHLSB | *Chlorocebus sabaeus* |
| A0A9B0WV75\|A0A9B0WV75_CHRAS | *Chrysochloris asiatica* |
| A0A9J7G3Z1\|A0A9J7G3Z1_CRIGR | *Cricetulus griseus* |
| A0A2Y9P1F4\|A0A2Y9P1F4_DELLE | *Delphinapterus leucas* |
| A0A1S3G4U8\|A0A1S3G4U8_DIPOR | *Dipodomys ordii* |
| A0A9L0JMD7\|A0A9L0JMD7_EQUAS | *Equus asinus* |
| A0A1S3AJR4\|A0A1S3AJR4_ERIEU | *Erinaceus europaeus* |
| A0A075IKA9\|A0A075IKA9_ERYPA | *Erythrocebus patas* |
| A0AA51YG73\|A0AA51YG73_FELCA | *Felis catus* |
| A0A075IMC2\|A0A075IMC2_9PRIM | *Gorilla gorilla* |
| G3QFY5\|G3QFY5_GORGO | *Gorilla gorilla gorilla* |
| A0A0P6JVW4\|A0A0P6JVW4_HETGA | *Heterocephalus glaber* |
| A0A8B7SND5\|A0A8B7SND5_HIPAR | *Hipposideros armiger* |
| **Q8IYV9\|IZUM1_HUMAN** | ***Homo sapiens*** |
| A0A8C5KP55\|A0A8C5KP55_JACJA | *Jaculus jaculus* |
| A0A075IMC7\|A0A075IMC7_LAGLA | *Lagothrix lagotricha* |
| A0A2U3YV44\|A0A2U3YV44_LEPWE | *Leptonychotes weddellii* |
| A0A340XT57\|A0A340XT57_LIPVE | *Lipotes vexillifer* |
| G3TPD8\|G3TPD8_LOXAF | *Loxodonta africana* |
| A0A2K5UGJ7\|A0A2K5UGJ7_MACFA | *Macaca fascicularis* |
| F6XH82\|F6XH82_MACMU | *Macaca mulatta* |
| A0A2K6C510\|A0A2K6C510_MACNE | *Macaca nemestrina* |
| A0A2K6ADS1\|A0A2K6ADS1_MANLE | *Mandrillus leucophaeus* |
| A0A1U7QS27\|A0A1U7QS27_MESAU | *Mesocricetus auratus* |
| A0A8B7FMH3\|A0A8B7FMH3_MICMU | *Microcebus murinus* |
| A0A8C6ALP5\|A0A8C6ALP5_MONMO | *Monodon monoceros* |
| A0A8C6CVS0\|A0A8C6CVS0_MOSMO | *Moschus moschiferus* |
| A0A6P5Q235\|A0A6P5Q235_MUSCR | *Mus caroli* |
| Q9D9J7\|IZUM1_MOUSE | *Mus musculus* |
| A0A8U0RYE2\|A0A8U0RYE2_MUSPF | *Mustela putorius furo* |
| G1P6T7\|G1P6T7_MYOLU | *Myotis lucifugus* |
| A0A8C6WAW6\|A0A8C6WAW6_NANGA | *Nannospalax galili* |
| A0A2Y9GFX2\|A0A2Y9GFX2_NEOSC | *Neomonachus schauinslandi* |
| A0A341DAB4\|A0A341DAB4_NEOAA | *Neophocaena asiaeorientalis asiaeorientalis* |
| G1QXF7\|G1QXF7_NOMLE | *Nomascus leucogenys* |
| A0A6P3FGL4\|A0A6P3FGL4_OCTDE | *Octodon degus* |
| A0A2U3WZY4\|A0A2U3WZY4_ODORO | *Odobenus rosmarus divergens* |
| A0A6J0X5E9\|A0A6J0X5E9_ODOVR | *Odocoileus virginianus texanus* |
| A0A8B7BBV6\|A0A8B7BBV6_ORYAF | *Orycteropus afer afer* |
| H0WN21\|H0WN21_OTOGA | *Otolemur garnettii* |
| W5PRD0\|W5PRD0_SHEEP | *Ovis aries* |
| A0A2R9A1Y2\|A0A2R9A1Y2_PANPA | *Pan paniscus* |
| H2QGS4\|H2QGS4_PANTR | *Pan troglodytes* |
| A0A8C8X5G7\|A0A8C8X5G7_PANLE | *Panthera leo* |
| A0A8C9KT86\|A0A8C9KT86_PANTA | *Panthera tigris altaica* |
| A0A6I9LU07\|A0A6I9LU07_PERMB | *Peromyscus maniculatus bairdii* |
| A0A6P5LNV6\|A0A6P5LNV6_PHACI | *Phascolarctos cinereus* |
| A0A8C9CFZ3\|A0A8C9CFZ3_PHOSS | *Phocoena sinus* |
| A0A6J2N838\|A0A6J2N838_9CHIR | *Phyllostomus discolor* |
| A0A455AS83\|A0A455AS83_PHYMC | *Physeter macrocephalus* |
| A0A8I5U5G3\|A0A8I5U5G3_PONAB | *Pongo abelii* |
| A0A8C8YD00\|A0A8C8YD00_PROSS | *Prolemur simus* |
| A0A2K6FJK7\|A0A2K6FJK7_PROCO | *Propithecus coquereli* |
| A0A6P6BLH4\|A0A6P6BLH4_PTEVA | *Pteropus vampyrus* |
| Q6AY06\|IZUM1_RAT | *Rattus norvegicus* |
| A0A671EGI0\|A0A671EGI0_RHIFE | *Rhinolophus ferrumequinum* |
| A0A2K6LQN1\|A0A2K6LQN1_RHIBE | *Rhinopithecus bieti* |
| A0A2K6NEL4\|A0A2K6NEL4_RHIRO | *Rhinopithecus roxellana* |
| A0A075IKB2\|A0A075IKB2_SAGLB | *Saguinus labiatus* |
| A0A2K6SYH0\|A0A2K6SYH0_SAIBB | *Saimiri boliviensis boliviensis* |
| A0A6J3HAJ3\|A0A6J3HAJ3_SAPAP | *Sapajus apella* |
| A0A287BMD8\|A0A287BMD8_PIG | *Sus scrofa* |
| A0A8D2G746\|A0A8D2G746_THEGE | *Theropithecus gelada* |
| A0A2Y9DWR9\|A0A2Y9DWR9_TRIMA | *Trichechus manatus latirostris* |
| A0A2U4APB7\|A0A2U4APB7_TURTR | *Tursiops truncatus* |
| A0A452SZ25\|A0A452SZ25_URSMA | *Ursus maritimus* |
| A0A6I9IF17\|A0A6I9IF17_VICPA | *Vicugna pacos* |
| A0A3Q7TIP7\|A0A3Q7TIP7_VULVU | *Vulpes vulpes* |

**Table S2. Known and predicted post-translational modifications of IZUMO1, hERp57, JUNO, and calnexin used for AlphaFold 3 predictions.** Related to Supplementary Figure S9.

| **Protein** | **Post-translational modification** |
| --- | --- |
| IZUMO1 (22-350) | 204N: N-Acetyl-beta-D-glucosamine  325S: Phosphoserine |
| hERp57 (25-505) | 61K: N6-Methyllysine  104K: N6-Acetyl-L-lysine  126S: Phosphoserine  129K: N6-Acetyl-L-lysine  146K: N6-Acetyl-L-lysine  152K: N6-Acetyl-L-lysine  163S: Phosphoserine  173K: N6-Acetyl-L-lysine  214K: N6-Acetyl-L-lysine  218K: N6-Acetyl-L-lysine  226K: N6-Acetyl-L-lysine  231K: N6-Acetyl-L-lysine  252K: N6-Acetyl-L-lysine  288K: N6-Acetyl-L-lysine  296K: N6-Acetyl-L-lysine  319T: Phosphothreonine  335K: N6-Acetyl-L-lysine  362K: N6-Acetyl-L-lysine  366K: N6-Acetyl-L-lysine  422K: N6-Acetyl-L-lysine  424S: Phosphoserine  433K: N6-Acetyl-L-lysine  456S: Phosphoserine  460K: N6-Acetyl-L-lysine  474S: Phosphoserine  478S: Phosphoserine  494K: N6-Acetyl-L-lysine |
| JUNO (20-250) | 73N: N-Acetyl-beta-D-glucosamine |
| calnexin (21-592) | 61K: N6-Acetyl-L-lysine  113K: N6-Acetyl-L-lysine  118K: N6-Acetyl-L-lysine  137K: N6-Acetyl-L-lysine  170K: N6-Acetyl-L-lysine  182K: N6-Acetyl-L-lysine  199K: N6-Acetyl-L-lysine  210K: N6-Acetyl-L-lysine  217K: N6-Acetyl-L-lysine  227K: N6-Acetyl-L-lysine  233K: N6-Acetyl-L-lysine  283K: N6-Acetyl-L-lysine  458K: N6-Acetyl-L-lysine  502C: S-Palmitoyl-L-cysteine  503C: S-Palmitoyl-L-cysteine  515K: N6-Acetyl-L-lysine  531K: N6-Acetyl-L-lysine  535K: N6-Acetyl-L-lysine  547K: N6-Acetyl-L-lysine  554S: Phosphoserine  562T: Phosphothreonine  564S: Phosphoserine  583S: Phosphoserine |
