# Supplementary material for: Sperm surface protein disulfide isomerase ERp57 is crucial for mammalian fertilization but functions independently of IZUMO1
Source: Cell Mol Biol Lett. 2026 Jun 27;31:149. doi: 10.1186/s11658-026-00984-y (PMC13599313; doi:10.1186/s11658-026-00984-y)
Supplement: Supplementary file 2 [file 11658_2026_984_MOESM2_ESM.docx]

**ADDITIONAL FILE 2 – Full Western Blot & Gel Images**

**Figure 1C Western blot**

Replicate 1

The red dashed-line box highlights the portion which was used in the figure.

**Additional Western blot Replicates for Figure 1C**

Replicate 2

Replicate 3

**Figure 3A Western blot**

Replicate 1

The red dashed-line box highlights the portion which was used in the figure.

**Additional Western blot Replicates for Figure 3A**

Replicate 2

Replicate 3

**Figure S1B Western blot**

Replicate 1

The red dashed-line box highlights the portion which was used in the figure.

**Additional Western blot Replicates for Figure S1B**

Replicate 2

Replicate 3

**Figure S1B protein gel**

Replicate 1

The red dashed-line box highlights the portion which was used in the figure.

*Coomassie-stained SDS-PAGE*

Replicate 2

*Coomassie-stained SDS-PAGE*

Replicate 3

*Coomassie-stained SDS-PAGE*

**Figure S3 nucleic acid gel**

*1^st^ panel: Parental (left), ERp57 fl/fl Stra8-cre (-/-)*

**Figure S3 nucleic acid gel**

*2^nd^ panel: Parental (right), ERp57 WT/WT Stra8-cre (+/+)*

**Figure S3 nucleic acid gel**

*3^rd^ panel: F1, ERp57 WT/fl Stra8-cre (+/-)*

**Figure S3 nucleic acid gel**

*4^th^ panel: F2, ERp57 fl/fl, somatic genotype*

**Figure S3 nucleic acid gel**

*5^th^ panel: F2, ERp57 fl/fl, sperm genotype (top)*

**Figure S3 nucleic acid gel**

*6^th^ panel: F2, ERp57 fl/fl, sperm genotype (bottom)*

**Figure S4 Western blots**

The red dashed-line boxes highlight the portions which were used in the figure.

**Figure S7A protein gel**

Replicate 1

The red dashed-line box highlights the portion which was used in the figure.

*Coomassie-stained SDS-PAGE*

Replicate 2

*Coomassie-stained SDS-PAGE*

Replicate 3

*Coomassie-stained SDS-PAGE*
